# Copper(II) Complexes with Carnosine Conjugates of Hyaluronic Acids at Different Dipeptide Loading Percentages Behave as Multiple SOD Mimics and Stimulate Nrf2 Translocation and Antioxidant Response in In Vitro Inflammatory Model

**DOI:** 10.3390/antiox12081632

**Published:** 2023-08-18

**Authors:** Francesco Bellia, Valeria Lanza, Irina Naletova, Barbara Tomasello, Valeria Ciaffaglione, Valentina Greco, Sebastiano Sciuto, Pietro Amico, Rosanna Inturri, Susanna Vaccaro, Tiziana Campagna, Francesco Attanasio, Giovanni Tabbì, Enrico Rizzarelli

**Affiliations:** 1 Institute of Crystallography, National Council of Research (CNR), P. Gaifami 18, 95126 Catania, Italy; francesco.bellia@cnr.it (F.B.); valeria.lanza@cnr.it (V.L.); irina.naletova@ic.cnr.it (I.N.); valeria.ciaffaglione@ic.cnr.it (V.C.); tiziana.campagna@cnr.it (T.C.); francesco.attanasio@cnr.it (F.A.); erizzarelli@unict.it (E.R.); 2 Department of Drug and Health Sciences, University of Catania, Viale Andrea Doria 6, 95125 Catania, Italy; btomase@unict.it; 3 Department of Chemical Sciences, University of Catania, Viale Andrea Doria 6, 95125 Catania, Italy; vgreco@unict.it (V.G.); ssciuto@unict.it (S.S.); 4 Fidia Farmaceutici SpA, Contrada Pizzuta, 96017 Noto, Italy; pamico@fidiapharma.it (P.A.); rinturri@fidiapharma.it (R.I.); svaccaro@fidiapharma.it (S.V.)

**Keywords:** copper, carnosine, hyaluronic acid, conjugates, antioxidant, SOD, Nrf2, osteoblast, inflammation

## Abstract

A series of copper(II) complexes with the formula [Cu^2+^Hy(*x*)Car%] varying the molecular weight (MW) of Hyaluronic acid (Hy, *x* = 200 or 700 kDa) conjugated with carnosine (Car) present at different loading were synthesized and characterized via different spectroscopic techniques. The metal complexes behaved as Cu, Zn-superoxide dismutase (SOD1) mimics and showed some of the most efficient reaction rate values produced using a synthetic and water-soluble copper(II)-based SOD mimic reported to date. The increase in the percentage of Car moieties parallels the enhancement of the I_50_ value determined via the indirect method of Fridovich. The presence of the non-functionalized Hy OH groups favors the scavenger activity of the copper(II) complexes with HyCar, recalling similar behavior previously found for the copper(II) complexes with Car conjugated using β-cyclodextrin or trehalose. In keeping with the new abilities of SOD1 to activate protective agents against oxidative stress in rheumatoid arthritis and osteoarthritis diseases, Cu^2+^ interaction with HyCar promotes the nuclear translocation of erythroid 2-related factor that regulates the expressions of target genes, including Heme-Oxigenase-1, thus stimulating an antioxidant response in osteoblasts subjected to an inflammatory/oxidative insult.

## 1. Introduction

Osteoarthritis (OA) and rheumatoid arthritis (RA) are the two main diseases that affect bone and cartilage [1]. RA is a chronic, inflammatory, and autoimmune disease that causes disability, systemic complications, and early death [2]. The etiopathogenesis of RA is characterized by synovial inflammation and synovial hyperplasia, ultimately leading to the destruction of bone and cartilage [3]. The pathogenesis of RA is multifactorial, and recent research calls into question the use of oxygen free radicals (ROS) as mediators of tissue damage together with inflammatory cytokines [4].

OA is a long-term chronic disease caused by the cartilage involved in joint alteration, which causes stiffness and swelling that often compromises the movement of joints and causes pain in elderly people [5]. Inflammation of different tissues, such as the synovial membrane, articular cartilage, and subchondral bone, represents the principal pathological effect of OA [6]. The dys-homeostasis of the redox state in the chondrocytes features OA due to excess production of ROS, including the superoxide radical anion O_2_^●−^ and hydroxyl radical (^●^OH). The high levels of ROS also induce an increase in pro-inflammatory cytokines, chondrocyte hyperplasia [7], and inflammatory responses [8], as well as lead to chondrocyte apoptosis [9].

Although the etiopathogenesis of these diseases is different, the two disorders share the cartilage target tissue and ROS alteration, while the multifactorial natures of these pathologies do not allow us to completely understand their etiology [10,11].

**Figure 1:**
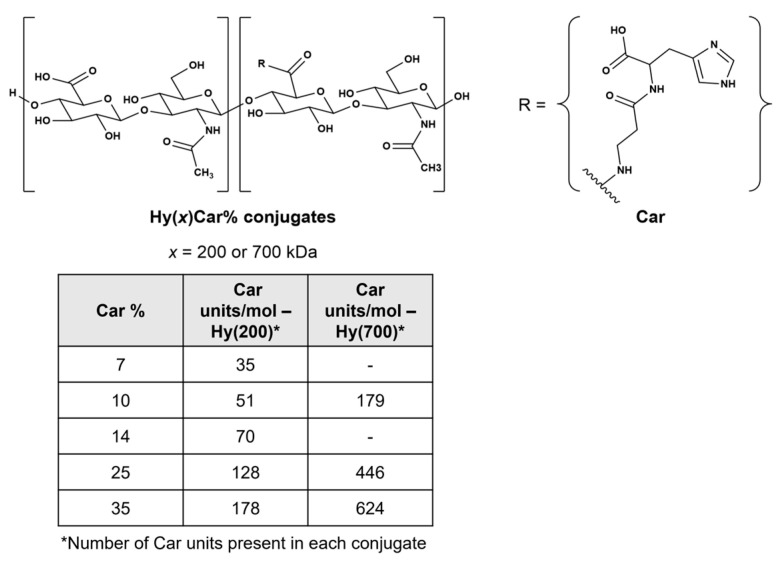
Chemical structures of HyCar conjugates. The *x* indicates the molecular weight of Hy in kDa, and Car% indicates the Car loading percentage. The Car units represent the average number of Car units conjugated to Hy.

Hyaluronic acid (Hy, Figure 1), which is a non-sulphated glycosaminoglycan that contains a disaccharide repeat structure [-4-d-glucuronic acid-β1-3-*N*-acetylglucosamine-β1-]*_n_*, is the main macromolecule of synovial fluid (SF) and a relevant component of proteoglycan aggregates within cartilage [12]. ROS degrade SF, fragment cartilage, and depolymerize hyaluronic acid, inducing viscosity loss and activating metalloproteases and bone resorption [13,14].

Carnosine (β-alanyl-L-histidine, Car, Figure 1) shows protective effects against oxidative stress induced by ROS, RNS, and RCS [15,16,17,18] by quenching these harmful species [19,20] both in vitro and in vivo [21,22,23,24]. Furthermore, Car inhibits hyaluronan degradation induction by ROS in vitro, improving the redox homeostasis in adjuvant arthritis in vivo [25]. However, the protective action of Car is drastically hampered by hydrolysis caused by the carnosinase enzymes [26,27,28]. Notably, the conjugation between Hy and Car impedes or delays the enzymatic hydrolysis of the dipeptide backbone and the degradation of the polymer by carnosinase and hyaluronidases, respectively [29]; this new molecular entity (HyCar) shows amyloid anti-aggregating ability [29] and antioxidant activity against ROS, RNS, and RCS [30]. Additionally, HyCar shows protective effects against both a collagen-induced arthritis (CIA) rat model, significantly reducing allodynia pain [31], and monosodium iodoacetate-induced (MIA) OA in rats, decreasing serum inflammatory cytokines [32]. Furthermore, the HyCar oral supplementation employed when using these experimental models of OA and RA represents an added value to improve a patient’s ability to cope with these degenerative diseases, though the mechanism of action is not entirely understood.

Different approaches are currently used to treat OA and RA by targeting ROS using superoxide dismutases (SODs). SODs are metalloenzymes present in the cytosol, the mitochondrial intermembrane space, and nucleus (Cu, Zn-SOD or SOD1); the mitochondrial matrix and inner membrane (Mn-SOD or SOD2) [33]; and the extracellular compartment (Cu, Zn-SOD or SOD3) [34]; their role as a major antioxidant agent is well recognized [35,36]. SODs act as ROS scavengers by transforming O_2_^●−^ into hydrogen peroxide (H_2_O_2_) and oxygen, protecting the redox state homeostasis in chondrocytes [37]. Furthermore, the levels of SOD2 significantly decrease in OA articular cartilage, suggesting the presence of excessive ROS levels in this tissue [38]. An increase in collagen-induced arthritis characterizes mice that are genetically deficient in SOD3 [39], while treatment with SOD3 inhibits cartilage and bone alteration in the CIA model [40].

The metalloenzyme Cu,Zn-SOD is recognized as the major regulator of antioxidant defense [34,35]. hSOD1 is also found in the nucleus acting as a transcription factor against oxidative stress [41,42]. As the use of the native enzyme as a pharmaceutical agent is hampered by low membrane permeability, immunogenicity, and rapid clearance [43], considerable efforts are currently being made to obtain the functional and structural mimics of SOD1.

Car forms different complex species with Cu^2+^ and Zn^2+^ [44], and its chelating ability provides different protective functions in in vitro and in vivo assays [45,46,47,48,49]. Copper(II) complexes mimicking SOD1 are thoroughly described in the literature [50,51]. Studies of copper(II) complexes with both linear and cyclic peptides [52,53,54,55,56,57,58,59] and functionalized cyclodextrins [60,61,62,63], including Car [64,65], describe the attempts to design efficient mimicking molecules. The aims of these studies were to obtain mimics endowed with stability, flexibility, adaptation to the different coordination geometries of the two copper redox states (Cu^2+^ and Cu^+^), and the ability to modulate the reduction potential required to perform superoxide dismutation.

Essential metal ions, such as Cu^2+^, are helpful to bones and joints at a physiological range of concentrations [66]. Different studies report that copper levels are significantly higher in the sera of patients with RA or OA than in those of healthy controls [67,68,69,70]. Furthermore, a positive correlation is reported between the serum copper level, the erythrocyte sedimentation rate, and morning stiffness. The increased metal concentrations negatively correlated with hemoglobin levels, which are auxiliary markers used in disease assessment [71,72]. With the increase in “free” Cu^2+^ content, the oxidation capacity of copper exceeds its own antioxidant capacity, thereby damaging joints [73]. Therefore, the design and synthesis of a ligand that both strongly binds Cu^2+^ and the metal complex of which mimics SOD1 provide an attractive opportunity to perform the dual function of an antioxidant.

In recent years, Hy has received much interest in Hy-bioconjugates; Hy systems containing covalently bound small molecules and drugs show improved solubility, prolonged half-lives, and increased targeting capabilities and bioavailability [74,75,76].

Extending our previous work on the synthesis and characterization of the conjugates of Hy with Car [77], we report on HyCar derivatives containing different Hy molecular weights (200 kDa and 700 kDa) and Car loading percentage values (from 7% to 35%) (Figure 1); these conjugates were used as chelating agents of Cu^2+^ to obtain new functional mimics of SOD1 [78,79]. Spectroscopic (FTIR-ATR, UV-vis, CD and ESR) experiments were performed to characterize the metal complexes; their SOD activity was assessed via the indirect assay designed by Beauchamp and Fridovich [35,80,81].

The expression of SOD is finely regulated by several transcription factors, such as the Nuclear Factor (NF)-κB, the specificity protein 1 (Sp-1), and the nuclear factor erythroid 2-related factor 2 (Nrf2) [82,83]. Nrf2 belongs to a family of basic leucine zipper transcription factors that bind to antioxidant response elements (AREs) that encode detoxifying enzymes and antioxidant proteins, as well as SODs [84]. Its translocation into the nucleus in response to increased intracellular ROS is mediated via the oxidation of the Kelch-like ECH-associated protein 1 (Keap1). Notably, the activation of the Keap1/Nrf2 pathway is suggested to be responsible for Mn-SOD’s mimic activities [85]. Furthermore, many reports have highlighted the ability of both Car [86,87,88] and Hy [89,90,91] to tune Nrf2 in different pathological conditions. Furthermore, several findings have shown a bidirectional interaction between copper and Nrf2 [92,93], the different effects of which have been attributed to copper concentration, cell type, disease pattern, and exposure to drugs or natural compounds [94,95,96,97,98,99,100]. Thus, we extended our work to ascertain the ability of HyCar to interact with the submicromolar copper present in the culture media and stimulate Nrf2 activity in human fetal osteoblastic cell line (hFOB) treated with the H_2_O_2_-conditioned medium of macrophages [101,102].

Our findings show that the copper(II) complexes with HyCar are very efficient SOD1 functional mimics in cell-free experiments, and their formation in in vitro assays protects cells from oxidative stress, inducing Nrf2 translocation to the nucleus, promoting the expressions of target genes, such as Heme-Oxigenase-1 (HO-1) and activating the Nrf2/HO-1 antioxidant pathway, which is a crucial response required for oxidative stress protection.

## 2. Materials and Methods

Commercially available reagents were purchased from Sigma-Aldrich (Milan, Italy), unless otherwise stated.

HyCar derivatives were synthesized via the method previously reported in [103]. The starting Hy MW was either 200 kDa or 700 kDa, and the final Car loading percentage ranged from 7 to 35%. The chemical conjugation between Hy and Car was proven via NMR studies, and other molecular information (molecular weight distribution and intrinsic viscosity) was previously reported [30].

### 2.1. Synthesis of Copper(II) Complexes and Hy(200)Car35%

The copper(II) complexes with Hy(200)Car35% were obtained by adding a solution of copper(II) nitrate to an aqueous solution of HyCar using a 1:1 copper to peptide molar ratio, taking into account the carnosine units present in Hy(200)Car35%. In addition, the copper(II) complexes with Hy and Car were synthesized using the same experimental approach. The pH was adjusted to either 7 or 9 through the addition of NaOH. The viscosity of the solution increased with the concentration enhancement of the starting HyCar derivative.

### 2.2. Infrared Spectroscopy (FTIR-ATR)

FTIR spectroscopy was used to characterize the complexes of Car, Hy(200), and Hy(200)Car35% with Cu^2+^. The spectra of freeze-dried samples were acquired in attenuated total reflection mode (ATR) using a Thermo Scientific Nicolet iS10 FT-IR Spectrometer. All spectra were recorded using 32 scans/spectrum at a resolution of 4 cm^−1^.

### 2.3. Electron Spin Resonance (ESR) Spectroscopy

A Bruker CW-ESR spectrometer (Elexsys E500) driven by Bruker XEpr program running under Linux, which is a Super-X microwave bridge (ER 049X) operating at 9.3–9.5 GHz, and a SHQE probe head were used during this study. An ER4131VT variable temperature unit was used to achieve the 150-kelvin temperature required to run all frozen solution ESR spectra. In order to increase the spectra resolution, a small amount of methanol (up to 10%) was added to copper(II) complex sample solutions. The resolution of the ESR spectra [104] was improved using an isotopically pure ^63^Cu(NO_3_)_2_ (0.05 M) to obtain the proper final concentration in each solution (4 × 10^−3^ M for L:Cu = 1:1; L = Hy(200)Car14% or Hy(200)Car35%), varying their pH values from 6.5 to 10).

In order to better determine the magnetic parameters, some of the experimental spectra were simulated via a revised version of the program Monoclin [105,106], which may produce values for overlapping species. Parallel spin Hamiltonian parameters were taken directly from the experimental spectra, and we always calculated them from the 2nd and 3rd lines to remove errors coming from second-order effects. Usually, initial estimates of the ESR parameters of a species were directly taken from the spectrum at a given pH after subtracting the values of the overlapping species derived from a spectrum only containing the latter species; the values obtained were, thus, used to reproduce the experimental spectrum obtained using pH values at which the two species coexist.

Instrumental settings of frozen solution ESR spectra were as follows: average number of scans, 4; microwave frequency, 9.421–9.429 GHz; modulation frequency, 100 kHz; modulation amplitude, 0.7 mT; time constant, 163 ms; sweep time, 2.8 min; microwave power, 20 mW; receiver gain, 60 dB.

### 2.4. UV-Vis and Circular Dichroism (CD) Spectroscopic Measurements

UV-vis spectra were recorded at room temperature with a UV-Vis-NIR Jasco V-670 spectrophotometer.

CD spectra of the copper(II) complexes using Hy(200)Car at different Car loading figures (35% and 14%) and a 1:1 Car/Cu^2+^ ratio were recorded at 25 °C under a constant flow of nitrogen via a JASCO 1500 spectropolarimeter at a scan rate of 100 nm min^−1^ and a resolution of 1 nm at different pH values. The 240–850-nanometer spectral range was covered using quartz cells with 1-centimeter path lengths. The results are reported as Δε (molar dichroic coefficient) in M^−1^ cm^−1^. The spectra were recorded as the average results of 5 scans. The calibration of the instrument was performed using a 0.06% solution of ammonium camphorsulfonate in water. All of the solutions were freshly prepared using double-distilled water; the concentrations of the ligands and the metal were the same as those employed for the UV-vis spectroscopy.

### 2.5. Superoxide Dismutase (SOD) Activity

The SOD activity was determined using the xanthine (Xa)/xanthine oxidase (XOD) system as a superoxide radical source and nitro blue tetrazolium chloride (NBT) as a detector molecule of O_2_^●−^ according to Beauchamp and Fridovich method [107]. The reduction in NBT was spectrophotometrically followed by monitoring the absorbance at 560 nm in either the presence or absence of the investigated complexes for 600 s. The I_50_ (the concentration that causes the 50% inhibition of the reduction in NBT) of the copper(II) complexes at pH 7.4 was determined. In brief, the mixture was prepared in phosphate buffer (10 mM, pH 7.4) by adding Xa (20 μM) and NBT (250 μM). The superoxide production was started by adding the proper amount of XOD able to determine a ΔA_560 of_ ~0.024. All measurements were carried out under stirring at 25 ± 0.2 °C using 1-centimeter path-length cuvettes. To rule out false positive results, a cross-check of the potential interference of the tested compounds on the Xa/XOD system was also carried out by following the uric acid production coming from Xa oxidation (295 nm). The measurements were performed in triplicate, and the concentration of the metal complexes ranged from 0.05 to 10 μM.

### 2.6. Cell Culture and Treatments

The immortalized human fetal osteoblast cell line hFOB 1.19 (hFOB, CRL-11372™) and murine monocyte/macrophage RAW 264.7 (ATCC TIB-71) cell were purchased from American Tissue Type Culture (ATCC). The copper content in the employed culture media added with different percentage of fetal bovine serum was previously reported (Cairns et al. manuscript in preparation), and those results are utilized in this study (see in brackets).

hFOB cells were maintained in DMEM and Ham’s F12 medium, which was supplemented with 10% fetal bovine serum (FBS), 2 mM of sodium pyruvate (Gibco, Waltham, MA, USA), 50 IU/mL of penicillin, and 50 µg/mL of streptomycin ([Cu^2+^] = 0.25 μM). For the culturing of RAW 264.7, Dulbecco’s modified Eagle’s medium (DMEM) with high glucose was used, along with 4 mM of Glutamax (Gibco, Waltham, MA, USA), 10% fetal bovine serum (FBS), 50 IU/mL of penicillin, and 50 µg/mL of streptomycin ([Cu^2+^] = 0.24 μM). All cells were cultured using a humidified incubator supplemented with 5% CO_2_ at 37 °C.

For the hFOB treatment, the cells were plated in medium with 1% FBS ([Cu^2+^] = 0.025 μM) and pre-treated for 1 h with Hy(200)Car35% (0.1% *w*/*v* in Hy(200) and 0.9 mM Car, which was reported as HyCar for the biological studies (0.9 mM), Hy (0.1% *w*/*v* Hy(200)), and a mixture of Hy and Car (Hy and Car). After 1 h, the hFOB cells were stimulated for either 20 or 48 h using macrophage-conditioned medium (MCM) (ratio 1:1 between DMEM/F12 with 1% FBS and DMEM with 5% FBS) harvested from macrophages pre-treated with 100 µM H_2_O_2_ for 24 h.

To prove the influence of Cu^2+^ on the biological effects of the tested compounds, the cells were also treated with 2,9-Dimethyl-4,7-diphenyl-1,10-phenanthroline disulphonic acid (BCS) (50 μM), which is an extracellular chelating molecule of Cu^2+^; BCS binds to the Cu^2+^ present in the culture medium, hindering the formation of the metal complex with Hy, Car, and HyCar in the in vitro assay.

### 2.7. Cells Proliferation Analysis

The changes in cell proliferation were quantified in hFOB cells through a label-free approach using the IncuCyte SX1 (Serial Number: IC 60068) live cell imaging system. hFOB cells were incubated with the compounds described earlier, and cell proliferation was assessed at 20 h. The phase contrast image was collected with 10× objective, and data were then analyzed using the Incucyte^®^ Artificial Intelligence (AI) Confluence Analysis Workflow, software v2022A (www.sartorius.com/en/products/live-cell-imaging-analysis/live-cell-analysis-software/incucyte-base-software), which quantifies cell surface area coverage as confluence values. The results were expressed as the proliferation rate between the control and treatments.

### 2.8. Oxidative Stress Analysis

Cellular oxidative stress was detected using the ROS probe 2′,7′-dichlorofluorescein diacetate (DCF-DA, 2.5 μM). hFOB cells were treated in two different 96-well plates, as previously described, DCF-DA was added at the end of treatment and incubated at 37 °C for 20 min, and DCF-DA fluorescence was monitored through live-cell imaging using the IncuCyte imaging system (Essen BioScience, Arbor Michigan, MI, USA) as a single endpoint measurement at 20 h after the addition of MCM.

The intensity of DCF fluorescence was analyzed via the IncuCyte^®^ Artificial Intelligence (AI) Confluence Analysis Workflow, Software v2022A (www.sartorius.com/en/products/live-cell-imaging-analysis/live-cell-analysis-software/incucyte-base-software) (Table 1 and Table 2) to quantify ROS levels as the total green fluorescence object area of each well.

### 2.9. Immunocytochemistry

To study Nrf2 localization after HyCar, Hy, Car, and Hy and Car mixture treatments, hFOB cells were seeded in 96 multiwells and treated for 48 h, as described in the cell culture section. For immunocytochemistry analysis, cells were fixed in 4% paraformaldehyde and permeabilized using 0.3% Triton X-100. Unspecific binding was blocked via 30 min of incubation in Dulbecco’s Phosphate-Buffered Saline with 0.2% gelatine. Nrf2 was detected by incubating overnight cells with rabbit anti-Nrf2 antibodies (code PA5-88084, dilution 1:200). After DPBS washing, cells were exposed for 1 h at room temperature to the secondary antibody (Goat anti-Rabbit IgG (H+L) Cross-Adsorbed Secondary Antibody, Alexa Fluor™ 488 A11008, dilution 1:500; Thermo Fisher, Waltham, MA, USA). Hoechst33342 (Molecular Probes, Eugene, OR, USA, 1 μg μL^−1^) was used to stain nuclear DNA. Images were analyzed under a Leica DMI 6000B epifluorescence inverted microscope using Adaptive Focus Control at 40× magnification. Images were taken at random locations throughout the area of the well for all of the samples. Sixty green (Nrf2)/blue (Hoechst33342) values were taken from twenty nuclear ROI (region of interest)/microscopic field, with three fields found per well. Images analysis after anti-Nrf2 immunostaining was carried out using ImageJ Software 1.53 g (http://imagej.nih.gov/ij (accessed on 6 August 2023); Java 1.8.0_112(64bit)). The ratio between nuclear and cytoplasmic fluorescence emissions was calculated.

### 2.10. Protein Lysate Preparation and Immunoblotting

Sample preparation and Western blot analysis were carried out according to the method described in a previous paper [108]. In brief, hFOB cells were collected vs centrifugation, and cell pellets were lysed in RIPA buffer containing a Halt Protease and Phosphatase Inhibitor Single-Use Cocktail for 30 min and centrifuged at 14,000× *g* for 10 min. The total protein amount was determined via Bradford’s method (Protein Assay Dye Reagent Concentrate, BioRad, Hercules, CA, USA). Equal amounts of proteins were separated using 4–20% Tris-Glycine gels (Bio-Rad, Hercules, CA, USA) and transferred onto nitrocellulose membranes. Proteins were detected using specific primary antibodies via incubation overnight at 4 °C. The antibodies used in protein detection were as follows: anti-SOD1 (Cat ab51254, 1:5000 dilution) and anti-HO-1 (Cat ab13248, 1:1000) derived from Abcam, (Waltham, MA, USA); anti-Actin (Cat#4970) derived from Cell Signaling Technology (Danvers, MA, USA). The appropriate infrared-dye-labelled secondary antibodies were used to detect primary antibodies. The secondary goat anti-rabbit (Cat# 925-32211) and goat anti-mouse (Cat# 926-68070) labelled with IRDye 800 (1:20,000) and IRDye 680 (1:20,000), respectively, were derived from LI-COR (Lincoln, NE, USA). The Odyssey Infrared Imaging System (LI-COR Biosciences, Lincoln, NE, USA) was used to scan the blot; quantitative densitometric analysis was performed using ImageJ (http://imagej.nih.gov/ij/ Java 1.8.0_112(64bit)). The results were expressed as arbitrary densitometric units (A.D.U.), and the values were normalized to Actin expression levels and presented as the percentage of untreated cells control.

### 2.11. Data and Statistical Analysis

Results were expressed as the mean ± standard deviation of at least two experiments using three biological replicates. Comparisons between all groups were analyzed using one-way analysis of variance (ANOVA) with Tukey’s multiple comparison test for post-hoc analyses, whereas the Student’s *t* test was used to compare the means between two groups either with or without BCS. GraphPad Prism 6.0 software was used to perform all statistical analyses (GraphPad Software Inc., La Jolla, CA, USA). Differences were considered to be significant at *p* values < 0.05.

## 3. Results and Discussion

### 3.1. HyCar Conjugates Offer Multi-Binding Sites to Cu^2+^

**Figure 2:**
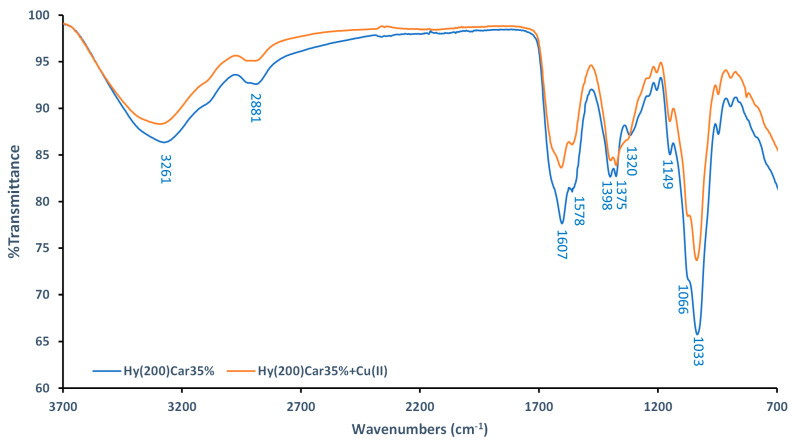
Comparison between FTIR−ATR spectra of Hy(200)Car35% (blue) and its complex with Cu^2+^ (orange).

FTIR-ATR spectra of Hy(200)Car35% and its complex with Cu^2+^ were recorded using dry samples. The spectra of the moieties that make up the conjugate (Hy(200), Car, and their copper(II) complexes) were also recorded (Figures S1 and S2), and they were in agreement with data previously reported in [109,110,111]. The spectra of Hy(200)Car35% show a broad band at 3261 cm^−1^ (Figure 2), which can be assigned to the hydroxyl groups of Hy, as reported for other Hy derivatives [112]. Different changes in the ligand spectra characterize the formation of the copper(II) complex using Hy(200)Car35% (Figure 2): (i) the decreased intensity of the band at 1607 cm^−1^ (stretching of the amide carbonyl of Car), (ii) the slight reduction in the bands’ intensities at 1398 cm^−1^ and 1375 cm^−1^ (stretching of COO^−^ and in-plane deformation of O-H, respectively), and (iii) the disappearance of the band at 1320 cm^−1^ (stretching of the imidazole ring). Moreover, significant reductions in the bands at 1149 cm^−1^, 1066 cm^−1^, and 1033 cm^−1^ corresponding to the vibrations in C–O–C, CO, and C–OH, respectively [109], were observed. However, further spectroscopic studies of the HyCar conjugate complex with Cu^2+^ were performed in aqueous solution to prove the metal binding details.

ESR and UV-vis spectroscopic measurements were carried out in the 6.5-to-10 pH range. The histidine imidazole ring present in the Car moiety is the preferred anchoring site for Cu^2+^ binding, but the metal ion interacting with HyCar experiences pH-dependent competition with a hydrogen bond network that involves the protonated imidazoles and the carboxylate residues, as previously reported for analogous systems containing the same residues [113]. This network, which can include the carbohydrate OH groups, contributes to the delay in the pH at which an appreciable amount of metal complex starts to form. Furthermore, the spectroscopic parameters of the HyCar copper(II) complex species (Table 3) appear to be significantly different to those reported for the analogous complexes formed using other Car conjugates [114,115,116], which were characterized by the binding of deprotonated amide nitrogen atoms. It is reasonable to attribute this feature of HyCar to the dense population of oxygen donor atoms, which can interact with the metal ion together with imidazole nitrogen, thus hindering the involvement of deprotonated amide nitrogen atoms.

**Figure 3:**
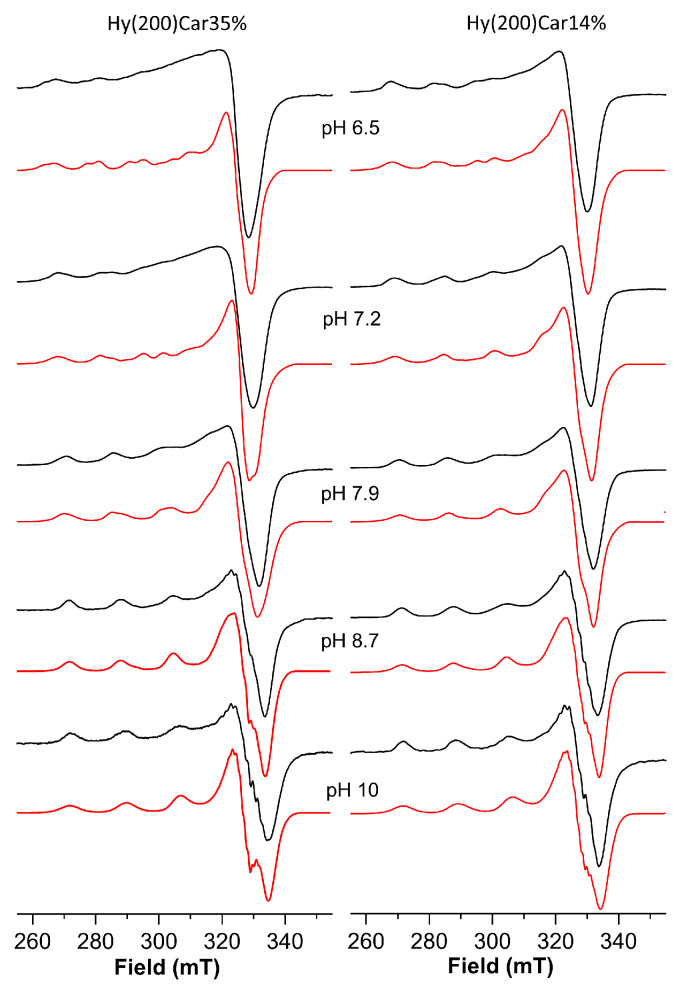
ESR spectra (150 K) of copper(II) complexes with Hy(200)Car35% and Hy(200)Car14% at different pH values. Simulated spectra are represented in red and experimental spectra are in black.

The Cu^2+^HyCar system at pH 6.5 shows the presence of two metal complex species. The simulation process of the ESR spectrum allow us to obtain the following Hamiltonian parameters: g_||_ = 2.369, A_||_ = 145 × 10^−4^ cm^−1^ and g_||_ = 2.335, A_||_ = 152 × 10^−4^ cm^−1^ (Figure 3 and Table 3). At the same pH value, the ESR spectrum of a solution containing Cu^2+^ and Hy200 shows a single species with the magnetic parameters g_||_ = 2.375, A_||_ = 145 × 10^−4^ cm^−1^, and a λ_max_ at 779 nm recalls those reported for the complex formed by copper(II) with glucuronic acid (g_||_ = 2.354, A_||_ = 144 × 10^−4^ cm^−1^ and a λ_max_ at 775 nm) [117]. These ESR parameters indicate that one of the species is characterized by a metal binding mode similar to that found for the copper(II) complex with Hy, which involves oxygen donor atoms alone. The second species shows lower g_||_ and higher A_||_ values, suggesting the presence of a stronger ligand field that can be attributed to the binding of the histidine imidazole nitrogen atom to Cu^2+^, in addition to the interactions between different oxygen donor atoms [118,119].

Upon increasing the pH value from 6.5 to 7.2, a λ_max_ blue-shift is observed, and another complex species forms that shows different magnetic parameters, i.e., g_||_ = 2.290, A_||_ = 166 × 10^−4^ cm^−1^, suggesting a stronger ligand field for the metal coordination sites. Such ESR parameters are typical of the coordination of at least two nitrogen atoms. Hence, the blue-shift can be attributed to a new chromophore arising from the binding of a further nitrogen atom, which occurs with the assistance of carboxylate oxygen atoms, and featuring by a {2N^Im^, 2O^COO^} coordination mode [120,121]. This metal complex becomes the predominant complex at a pH value of 7.9, where another complex was also found. This minor species becomes the only complex present at a pH value of 8.7, which is characterized by the magnetic parameters g_||_ = 2.273 and A_||_ = 172 × 10^−4^ cm^−1^; the λ_max_ value and the magnetic parameters suggest a {3N^Im^, O^COO^} coordination mode. Further support comes from the observation of similar ESR Hamiltonian parameters, which found for Cu^2+^ coordinated by vinylimidazole copolymers [122]. Finally, at very basic pH values, the main species showed g_||_ = 2.258 and A_||_ = 184 × 10^−4^ cm^−1^ values, implying the binding of a further histidine imidazole nitrogen atom, as was found in similar systems [54,103].

The representative CD spectra of Cu^2+^Hy(200)Car35% system are reported in Figure S3. These spectra show negative CD bands in the 600–800-nanometer region. In particular, at pH 5.0, a band exhibiting a minimum around 750 nm can be assigned to the copper d-d transition, which shifted toward a shorter wavelength (620 nm) as the pH value was brought to 8.5. This observed blue shift and the associated decrease in the Δε values starting from pH 6.6 indicate a stronger ligand field, which is in accordance with the ESR and visible spectral parameters (Table 4). In addition, positive bands at about 260 nm are found in the far UV spectral range; these bands arise from the π2 imidazole to Cu^2+^ charge transfer (CT) transition, whereas the negative bands occurring at around 330 nm can be assigned to the π1 imidazole to Cu^2+^ CT transition.

The well-resolved spectra of the copper(II) complexes with HyCar at the highest Car loading were difficult to obtain due to the aqueous solution’s high viscosity, which decreases when the Car loading is reduced. The I_50_ value for different Cu^2+^Hy(200)Car derivatives reaches an efficiency plateau at 14% of carnosine loading (vide infra); therefore, spectroscopic measurements were carried out using the Cu^2+^-Hy(200)Car14% system.

The spectrum of the frozen solution of this system at pH = 6.5 (Table 4, Figure 3) shows a main species characterized by g_||_ = 2.335, A_||_ = 152 × 10^−4^ cm^−1^ values, which indicate Cu^2+^ bound to a 1N^Im^, 1O^CO^ set of donor atoms; these atoms bind differently to those of the Cu^2+^Hy(200)Car35% system at the same pH value, while the other coexistent species contains the metal ion involved in a 2N^Im^,2O^COO^ coordination environment (g_||_ = 2.290, A_||_ = 166 × 10^−4^ cm^−1^).

Upon raising the pH value to 7.2, these two species still coexist, but their relative amounts were almost reversed, with the metal complex characterized by 2N^Im^, 2O^COO^ binding mode being the most abundant, before becoming the only species present at pH = 7.9.

At pH 8.7, only a metal complex was found characterized by the g_||_ = 2.273 and A_||_ = 172 × 10^−4^ cm^−1^ values, which suggests the further involvement of imidazole nitrogen donor atom; this species survives until pH 10, at which point another copper(II) complex forms, the magnetic parameters (g_||_ = 2.258 and A_||_ = 184 × 10^−4^ cm^−1^) and λ_max_ value (677 nm) of which indicate a 4N binding mode.

CD measurements were also performed via the Cu^2+^-Hy(200)Car14% system (Figure S4). The CD spectra show a trend that parallels that found for the Cu^2+^-Hy(200)Car35% system and is in keeping with the ESR data.

It is interesting to note that the simulation process required to obtain the ESR parameters indicates that the copper(II) complex with Hy(200)Car14% forms a higher percentage of the species featured using a 2N^Im^, 2O^COO^ set of donor atoms at physiological pH, in comparison to that found for the analogous species containing the ligand Hy(200)Car35%. Hy(200)Car14%, on average, possesses a derivatized disaccharidic unit every seven repetitions, whereas for Hy(200)Car35%, this value drops to three, conferring to the latter derivative a higher “stiffness” of the polymeric chain and giving rise to a very crowded system. This difference might favor the interaction between Cu^2+^ and the histidine and carboxylate residues present in two different disaccharide units in the more flexible Hy(200)Car14%.

### 3.2. Copper(II) Complexes with HyCar Show a High O_2_^●−^ Scavenging Ability

The scavenging behavior of the copper(II) complexes with HyCar at different Hy MW and Car loading was comparatively evaluated by determining the SOD-like activity. The copper(II) complexes with HyCar show a dose-dependent response, which is similar to those of most of the SOD mimetics [123,124,125,126,127,128].

Cu^2+^Hy(200) and Cu^2+^Hy(700) show a SOD-like activity similar to that displayed by Cu^2+^Car (Table 5), and their scavenging abilities are comparable to that found for the copper(II) complex with the phosphate ligand (Table 5) [65,115].

**Figure 4:**
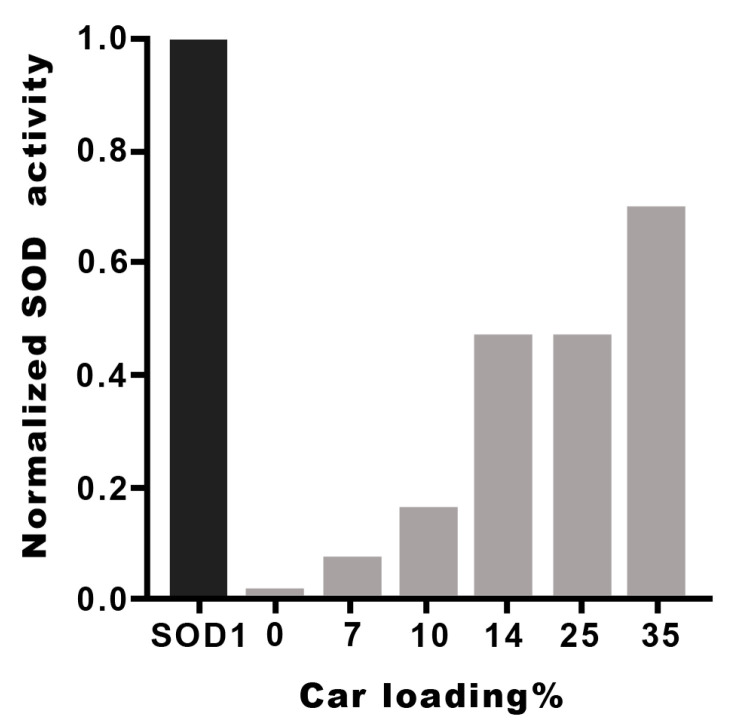
SOD1-like activity of Cu^2+^Hy(200)Car complexes at different Car loading percentages. The I_50_ values of different HyCar conjugates were normalized in respect to the I_50_ value of SOD1 [65].

The I_50_ and k_cat_ values of the metal complexes with the different Hy(200)Car conjugates indicate that the new molecular entities possess scavenging capacities comparable to that found by the native SOD1. The metal complexes with HyCar increase their SOD1-like ability in proportion to the enhancement of Car loading, as shown in Figure 4 for Hy(200)Car conjugates. This trend is more evident for copper(II) complexes with the Hy(200) conjugates than for those with the Hy(700) derivatives. The conjugates of Hy at lower molecular weights (200 kDa) are more efficient with respect to the corresponding conjugates at higher molecular weight (700 kDa), which is probably due to the higher viscosity of the Hy(700) solutions.

Previously, the I_50_ values of copper(II) complexes with histidine containing dipeptides, as well as their conjugates with a disaccharide, α,α-trehalose, cyclic oligosaccharide, or β-cyclodextrin, have been reported (Table S1), showing that the saccharide conjugation improves the SOD-like activity of the simple peptide metal complexes [65,115]. The increased scavenging capacity indicates the contribution of OH residues, which are present in the saccharides and promote the binding of the superoxide anion radical via the formation of H-bonds.

Analogously, the high SOD-like capacity of the copper(II) complexes with HyCar derivatives can be reasonably ascribed to the contribution of the Hy scaffold to the interactions between the O_2_^●−^ species.

**Figure 5:**
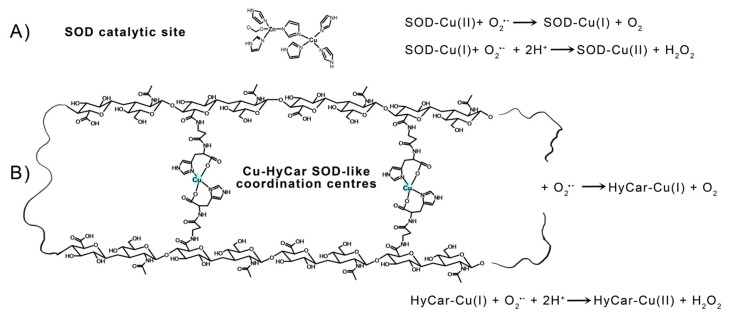
Catalytic cycle of SOD1: (**A**) a tentative structure of multiple copper(II) binding sites in Cu-HyCar, and (**B**) a hypothetical (but based on our results) scheme detailing the O_2_^●−^ scavenger abilities of copper(II) complexes with HyCar.

The electrostatic loop formed by charged (Lys, Arg, Asp, and Glu) and polar (Asn, Ser, and Thr) residues gives rise to a positive electric field at the entrance to the channel leading towards the catalytic metal center in mature SOD1. These residues enhance the enzyme function by providing an electrostatic path to anionic superoxide radical toward solvent-accessible catalytic Cu^2+^ in the active site [129]. Furthermore, point mutation findings demonstrate that the specific folding and orientation of the electrostatic loop, as well as the site and identity of charged residues within it, strongly affect its capacity to enhance catalytic activity [130]. The hydroxyl residues of Hy can give rise to a network of hydrogen bonds with the radical anion superoxide, favoring the interaction with the catalytic metal center and imitating the role of the above-cited positive-charged amino acids present in the native SOD1. In addition to this effect, which is also known as “electrostatic tunnelling” [131], the efficiency of SOD1 functional mimetics has been correlated based on the flexibility of the ligand, which would allow the binding donor atoms to be rearranged around the metal center along the catalytic pathway; this process can favor the pseudo-octahedral Jahn–Teller distorted Cu^2+^ reduction in the tetragonal Cu^+^. A rough measure of this flexibility can be provided by the Peisach–Blumberg method, which provides an indication of the tetrahedral distortion tendency of a metal complex based on the g_||_/A_||_ ratio calculation [132,133]. The ratio value of 138, together with the weak ligand field around the Cu^2+^ center revealed using the EPR and UV-vis parameters indicate a low stiffening/rigidity in the coordination geometry of the active metal complex species. Thus, it favors the redox process that does not require a high energy contribution to the change in coordination geometry—change that assists the variation in Cu^2+^ to the Cu^+^ oxidative state. This binding mode of HyCar represents a new ligand flexibility that, at the same time, generates polynuclear copper(II) complexes as Cu,Zn-SOD active site models (Figure 5), the number of which increases as the Car loading percentage enhances. To the best of our knowledge, Cu^2+^HyCar complexes are a new and unique class of multiple-site SOD1 mimetics, which employ this feature to reach a scavenging ability comparable to that of the native enzyme.

Hy is employed in OA and other joint injuries, mainly in its high molecular weight form, while the low molecular weight form of Hy, or its fragments, could be provided through oral administration because they are more easily absorbed [134,135]. Though the antioxidant ability of Hy has been proven [136], on the other hand, the ability of ROS to degrade Hy represents one of the metabolic pathways used to carry out degradation in the body by randomly cleaving side groups from Hy chains [137]. This potential incongruity has recently been resolved based on the finding that SOD3 directly binds to Hy in vitro and in vivo, inhibiting ROS-induced fragmentation of Hy [138]. In this context, it appears reasonable to regard our SOD mimetic as a Hy protective agent based on the superoxide insult that contributes to the oxidative stress that is a feature of OA and joint diseases.

Recently, some additional functions of hSOD1 related to its conventional role in superoxide radical scavenging have been reported, proving its role in redox signaling [139]. This development prompted us to verify the copper (II) complex based on the HyCar’s ability to show additional protective effects against ROS insults in some in vitro assays, as we were aware of the Nrf2 antioxidant defense system’s involvement in human osteoarthritis and bone degeneration [140,141].

### 3.3. HyCar Promotes Proliferation of MCM-Stressed hFOB Cells Interacting with Cu^2+^

The important role of Cu^2+^ and HyCar, as well as its moieties Hy and Car, in the regulation of cell viability and growth was studied using the osteoblast cell model hFOB under inflammatory stress, monitoring cell proliferation’s role as a marker of cell resistance during inflammatory processes. This stressed status was induced via MCM treatment. Numerous reports highlight the role of the physiological amount of copper in modulating cell proliferation [142,143,144], as well as the well-known abilities of individual substances, i.e., Hy and Car, in regulating this process [145,146]. HyCar, Car, and Hy have decreasing affinity to Cu^2+^ binding, and this different capacity can affect their interaction with the metal ions present in complete culture media. BCS is a metal competitive ligand with a copper binding affinity higher than those of HyCar, Car and Hy, and it decreases their interaction with Cu^2+^ in inverse proportion to their metal affinities.

**Figure 6:**
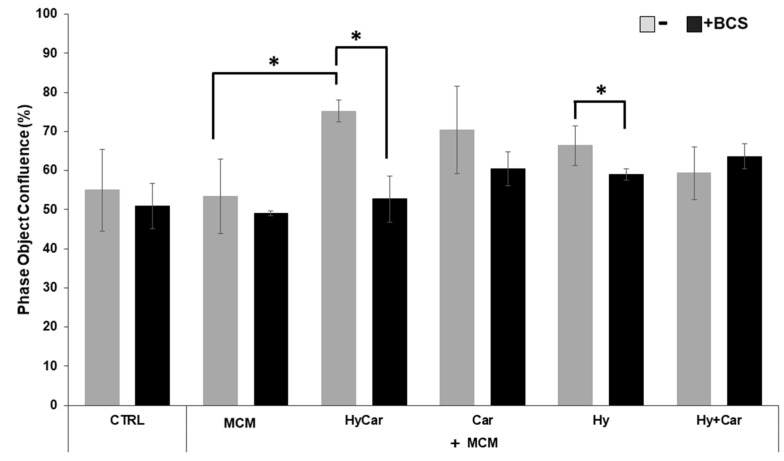
Cu^2+^’s interactions with Car, Hy, and HyCar stimulate hFOB proliferation under MCM stress. Cells were pre-treated with HyCar, Hy, Car, or Hy and Car in the presence or absence of BCS (50 μM) for 1 h and exposed to MCM for 20 h. After the treatments, the plates were imaged via the IncuCyte instrument. Total occupied area of hFOB cells was calculated via IncuCyte Base Analysis using the “Artificial Intelligence (AI)” mask for cell detection; for other parameters, refer to Table 1 in the Section 2. The instrument software generated the percentage of cell confluence (as indicated in the Section 2) for each well. The data points and error bars represent the mean ± SD of two experiments in quadruplicates. Statistical significance is indicated as * *p* < 0.05.

No overt effects of MCM on hFOB cell proliferation were noted. Instead, HyCar, Car, and Hy treatment stimulates cell proliferation in the presence of MCM, but only HyCar does so significantly (Figure 6 and Figure S5A). Indeed, HyCar exposure induces the highest proliferation rate, reaching the 75% ± 3 confluence in the first 20 h, relative to that of the MCM control cells (approximately 53% ± 9 confluence). No significant effects of BCS on the proliferation of both control and MCM-treated cells were noted in our results. Conversely, BCS’ addition to MCM-treated hFOB cells affects the induction of cell proliferation by HyCar (53% ± 5 confluence), which is suppressed, while the other compounds show less evident or significant inhibition. Similarly, the use of HyCar alone bolsters hFOB proliferation, whereas the proliferation rates of hFOB cells treated with other compounds were quite comparable to those of control cells. The effects of BCS addition are evident only in HyCar-treated cells, with a modest significant reduction in cell proliferation being noted (Figures S5B and S6A).

Generally, hFOB cells are spindle shaped and elongated, with only slight areas of spreading at the ends of long lamellipodia [147]; HyCar treatment generates a uniform elongated shape and parallel orientation, with cells overlapping each other due to rapid cell multiplication (Figure S5A,B), while some MCM-treated cells became round. After exposure for 48 h, all cells achieved 100% confluence and a uniform elongated shape. Therefore, the observed cell morphology after 20 h of molecule treatment supports the ability of HyCar to promote osteoblast proliferation more successfully than the single component of the conjugate.

Together, these results indicate that HyCar interaction with copper present in the culture medium markedly stimulates proliferation in an inflammatory and oxidative environment, while the BCS chelation of the sub-micromolar copper present in the culture medium slows down this cellular process. This finding highlights the role played by the copper(II)-HyCar complex formation in sustaining cell proliferation. This effect is shared with the other assayed molecules to a lesser extent, which is in keeping with their different affinities with copper.

### 3.4. Cu^2+^’s Interaction with the Conjugate and Its Components Reduces MCM-Induced Oxidative Stress

**Figure 7:**
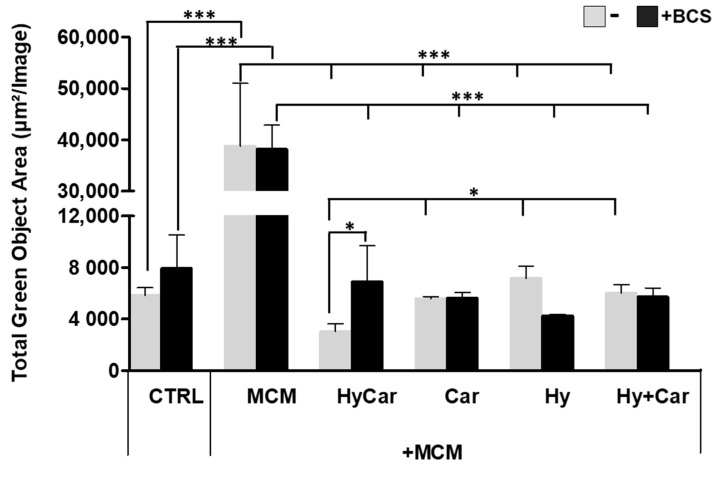
Hy, Car, and their conjugate interaction with Cu^2+^ present in culture medium reduce ROS levels in hFOB cells stressed with MCM. hFOB cells were pre-treated with HyCar, Hy, Car, or Hy and Car in the presence or absence of BCS (50 µM) for 1 h and exposed to MCM for 20 h. Fluorescence measurement was performed via the IncuCyte system using the “Artificial Intelligence (AI)” mask for cell detection; for other parameters, refer to Table 2 in the Section 2. Data are expressed as the total green fluorescence area of cells per image. Statistical significance is indicated as * *p* < 0.05 or *** *p* < 0.001.

hFOB cells were subject to insults via MCM treatment to mimic oxidative stress and inflammation in the in vitro assay; the resulting ROS production was then examined in the absence or presence of all assayed compounds. Notably, the exposure to MCM-induced a significant increase in ROS production, whereas HyCar (3037.6 μm^2^/Image ± 600) and all tested compounds displayed a marked reduction in oxidative stress (Figure 7 and Figure S7A). The addition of BCS affects ROS production in the untreated control, while its effect does not appear to be significant in MCM-stressed cells. BCS treatment induces a decrease in antioxidant activity, which is particularly evident for HyCar (6925.6 μm^2^/Image ± 2769), thus diminishing its protective effect. In addition, HyCar and Car treatment alone are able to reduce the physiological content of ROS in hFOB cells. Conversely, Hy positively affects ROS levels, whereas no alterations in ROS production were observed using the Hy and Car mixture. Finally, BCS’ addition to the compound has no effects on ROS levels (Figures S6B and S7B).

Overall, the conjugate shows a synergic antioxidant effect comparable to that displayed by its moieties, the ability of which to reduce ROS levels has previously been reported for Car [148,149] and Hy [150] in both in vitro and in vivo assays. Furthermore, despite the low metal concentrations present in the culture medium, Cu^2+^’s interaction with HyCar, Car, and Hy drives the antioxidant effect in a way that recalls the different metal affinities of Car [151] and Hy [152]. The impact on ROS production induced via the addition of the competitive copper chelator BCS supports the metal’s involvement in HyCar activity.

### 3.5. Cu^2+^–HyCar Interaction Markedly Induces Nrf2 Nuclear Localization and Nrf2/HO-1 Pathway Activation in MCM-Treated hFOB Cells

Nuclear factor erythroid 2-related factor 2, as it is a basic redox-sensitive leucine zipper (bZIP) transcription factor, protects against inflammation [153] and plays a marked role in intracellular antioxidant defense and phase II detoxification reaction through the upregulation of various antioxidant responsive genes [154,155].

**Figure 8:**
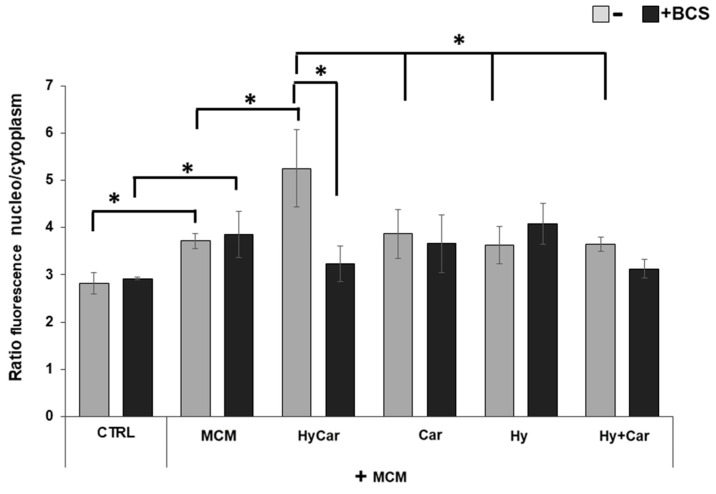
The nuclear localization of Nrf2 in MCM-stressed hFOB cells is significantly promoted by HyCar in a Cu^2+^-dependent mode. hFOB cells were pre-treated with HyCar, Hy, Car, or Hy and Car in the presence or absence of BCS (50 µM) for 1 h and exposed to MCM for 48 h. Ratio of fluorescence intensity between nucleus and cytoplasm represents the Nrf2 expression level. All values are mean ± SD of five random field images (see Figure S7) derived from three independent experiments. Significant differences were indicated using * *p* ≤ 0.05.

The immunofluorescence results (Figure 8 and Figure S9) show that human osteoblasts exposed to MCM moderately increase the nuclear localization of the Nrf2 (3.7 ± 0.1) in response to the increase in ROS levels relative to those of control cells (2.8 ± 0.2). Treatment with HyCar promotes a marked nuclear translocation of Nrf2 in MCM-stressed hFOB cells (5.2 ± 0.8). The addition of Car, Hy, and the mixture of Car and Hy in the presence of MCM does not significantly induce Nrf2 movement into the nucleus (3.9 ± 0.5, 3.6 ± 0.4 and 3.6 ± 0.2, respectively). Interestingly, the chelation of the submicromolar Cu^2+^ present in culture medium by the added BCS drastically reduces the HyCar-induced nuclear localization of Nrf2 (38.5%, 3.2 ± 0.4), whereas the other compounds are not significantly affected by BCS. Unlike MCM-stressed hFOB, no effects on Nrf2 translocation are observed in hFOB cells treated with compounds alone or in the presence of BCS (Figures S8 and S10).

**Figure 9:**
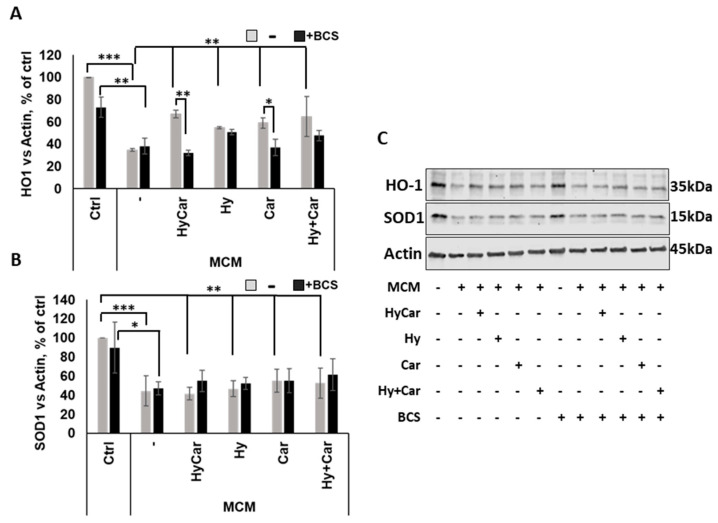
HyCar affects the Nrf2 signaling pathway in MCM-stressed hFOB cells in a copper-dependent manner. Densitometric analysis (**A**,**B**) and representative Western blot images (**C**) of HO-1 and SOD1 expression in hFOB pre-treated with HyCar, Hy, Car, or Hy and Car in the presence or absence of BCS (50 µM) for 1 h and exposed to MCM for 48 h. The protein expression levels are reported as the ratio over actin. Data are expressed as mean ± SD. Significant differences were indicated using the following methods: * *p* ≤ 0.05, ** *p* ≤ 0.01, *** *p* ≤ 0.001.

In response to oxidative stress, Nrf2 activation via translocation into the nucleus regulates the expression of target genes, including HO-1 and SOD1 [154]. Our data demonstrate that MCM insult depresses the Nrf2 signaling pathway by drastically reducing HO-1 expression. This effect is largely counteracted via pre-treatment with all compounds, which raise the intracellular HO-1 levels. Interestingly, the copper depletion by BCS markedly blocks the activation of this antioxidant pathway in HyCar- and Car-pre-treated cells (Figure 9A,C). Among the single compounds, only HyCar treatment is able to upregulate HO-1 expression, and its effects are reversed by BCS (Figure S11A,C). The results of the present study are consistent with the literature, which clearly reports the protective effects of Hy [91,156] and Car [157,158] against oxidative stress injury and copper involvement in Nrf2/HO-1 activation [159]. In addition, as expected, SOD1 expression is inhibited by MCM stress, but no effects occur after pre-treatment with all compounds with or without BCS at 48 h (Figure 9B,C). Notably, HyCar strongly stimulates SOD1 expression (Figure S11B,C) when added to cells alone, and BCS downregulates intracellular SOD1 content in HyCar-, Car-, and Hy-treated cells. Based on this result, along with other various pieces of evidence, such as current data regarding SOD mimic proprieties and ROS reduction at 20 h, as well as our recent results regarding SOD1 intracellular total levels at 5 h, we can reasonably suggest a biphasic response over time for SOD1. This behavior would be characterized by an early and short antioxidant cell response in terms of total SOD expression and activity, which efficiently protects against oxidative milieu, followed a long period of steady state with no active role for SOD1.

These results suggest that the effect of HyCar on the MCM-stressed hFOB is not exclusively caused by the chemical properties of the HyCar as a SOD mimetic, but also occur through biological mechanisms and the activation of intracellular signal transduction.

## 4. Conclusions

Overall, our findings indicate that HyCar is a ligand capable of providing both Cu^2+^ multiple binding sites, which rise with increased dipeptide loading, and an OH residue network, which entraps the O_2_^●−^ in the right position to interact with the metal center. These features allowed us to obtain a functional SOD1 mimic with a I_50_ value in the same order of magnitude as that of the native enzyme.

Furthermore, HyCar interaction with submicromolar Cu^2+^ contained in culture medium favors the Nrf2′s translocation to the nucleus, giving rise to a protective activity against the oxidative stress induced in hFOB cells treated with inflammatory MCM in an in vitro assay. This study demonstrates that the protective effects against the oxidative stress injury are achieved through the activation of Nrf2-signaling pathways connected to antioxidant responses mediated via the activation of HO1 and the modulation of SOD1.

Finally, the evidence reported here regarding the way in which HyCar induces its copper-mediated inhibition of ROS production suggests a new potential contribution of the molecule in counteracting the inflammatory and oxidative stresses found in previous MIA and CIA studies [31,32].

## Figures and Tables

**Table 1:** Analysis of cell confluence. Selected parameters used to set the base analysis with the “Artificial Intelligence (AI)” mask.

Segmentation adjustment	AI confluence
Cleanup	0
Area	Minimum 500.00 μm^2^
Image channels	Phase
Magnification	10×
Segmentation adjustment	AI confluence

**Table 2:** Analysis of green fluorescence. Selected parameters used to set the fluorescence analysis mask for the DCF probe.

Segmentation adjustment	AI confluence
Cleanup	0
Area	Minimum 500.00 μm^2^
Green Segmentation	Surface fit
Threshold (GCU)	2000
Edge Sensitivity	100
Image channels	Phase and green
Magnification	10×

**Table 3:** Spectroscopic parameters of the copper (II) complex species formed by Hy(200)Car35% at different pH values.

pH	g_||_	A_||_ (×10^4^ cm^−1^)	λ_max_ (nm)	ε (M^−1^ cm^−1^)
6.5	2.369	145	745	55
	2.335	152		
7.2	2.335	152	730	60
	2.290	166		
7.9	2.290	166	682	60
	2.273	172		
8.7	2.273	172	680	52
10.1	2.273	172		
	2.258	184	662	62

**Table 4:** Spectroscopic parameters of copper (II) complex species formed by Hy(200)Car14% at different pH values.

pH	g_||_	A_||_ (×10^4^ cm^−1^)	λ_max_ (nm)	ε (M^−1^ cm^−1^)	Coordination Mode
6.5	2.335	152	742	58	N^Im^, O^CO^
	2.290	166			2N^Im^, 2O^COO^
7.2	2.335	152	730	62	N^Im^, O^CO^
	2.290	166			2N^Im^, 2O^COO^
7.9	2.290	166	714	62	2N^Im^, 2O^COO^
8.7	2.273	172	684	65	3N^Im^, (O^COO^)
10.1	2.273	172			3N^Im^, (O^COO^)
	2.258	184	677	64	4N^Im^

**Table 5:** I_50_ and k_cat_ values of the following elements: (i) the copper(II) complexes with Hy(200)_,_ Hy(700), or Car; (ii) the copper(II) complexes with HyCar ligands of Hy and different MW and Car loading; and (iii) SOD1 and copper(II) complexes with phosphate.

SOD1/SOD1 Mimic	I_50_ μM	K_cat_ (×10^8^)
SOD1	0.014(3)	15(3)
Cu^2+^Hy(700)Car 10%	0.05(4)	4.2(3)
Cu^2+^Hy(700)Car 25%	0.05(2)	4.2(2)
Cu^2+^Hy(700)Car 35%	0.09(2)	1.9(2)
Cu^2+^Hy(200)Car 7%	0.21(4)	1.0(3)
Cu^2+^Hy(200)Car 10%	0.09(1)	2.4(4)
Cu^2+^Hy(200)Car 14%	0.03(2)	7.1(3)
Cu^2+^Hy(200)Car 25%	0.03 (2)	7.1(4)
Cu^2+^Hy(200)Car 35%	0.02(1)	11(3)
Cu^2+^Hy(700)	0.8(2)	0.3(2)
Cu^2+^Hy(200)	1.0(3)	0.2(3)
Cu^2+^Car	1.0(2)	0.2(4)
CuHPO_4_ ^a^	1.06	-

^a^ Data taken from Ref. [65].

## Data Availability

Data are contained within the article and Supplementary Files.

## Supplementary Materials

The following supporting information can be downloaded via the following link: https://www.mdpi.com/article/10.3390/antiox12081632/s1, Figure S1: Comparison between FTIR-ATR spectra of Car and its complex with Cu^2+^; Figure S2: Comparison between FTIR-ATR spectra of Hy(200) and its complex with Cu^2+^; Figure S3: UV-vis CD spectra for the Cu^2+^Hy(200)Car35% system at different pHs; Figure S4: UV-vis CD spectra for the Cu^2+^Hy(200)Car14% at different pHs. Table S1: I_50_ values (indirect method assay) of copper(II) complexes with histidine containing dipeptides and their glycosidic conjugates in phosphate buffer; Figure S5A,B: Representative image acquired via the IncuCyte system in brightfield of hFOB cells pre-treated with HyCar, Hy, Car, or Hy and Car in the presence or absence of BCS; Figure S6A,B: Effects of Hy, Car, and their conjugate interaction with Cu^2+^ present in culture medium on cell proliferation and ROS levels in hFOB cells; Figure S7A,B: Representative images acquired via the IncuCyte system in brightfield and fluorescence modes of hFOB cells pre-treated with HyCar, Hy, Car, or Hy and Car in the presence or absence of BCS; Figure S8: Effects of Hy, Car, and their conjugate interaction with Cu^2+^ present in culture medium on nuclear localization of Nrf2 in hFOB; Figures S9 and S10: Representative fluorescence image of the nuclear and cytoplasmic expression of Nrf2 in hFob cells; Figure S11: HyCar affects Nrf2 signaling pathway in hFOB cells in a copper-dependent way.
