# Supplementary material for: Copper(II) Complexes with Carnosine Conjugates of Hyaluronic Acids at Different Dipeptide Loading Percentages Behave as Multiple SOD Mimics and Stimulate Nrf2 Translocation and Antioxidant Response in In Vitro Inflammatory Model
Source: Antioxidants (Basel). 2023 Aug 18;12(8):1632. doi: 10.3390/antiox12081632 (PMC10452038; doi:10.3390/antiox12081632)
Supplement: Supplementary file 1 [file antioxidants-12-01632-s001.zip › antioxidants-2525380-supplementary.pdf]

Copper(II) Complexes with Carnosine Conjugates of Hyaluronic Acids at Different Dipeptide Loading Percentages Behave as Multiple SOD Mimics and Stimulate Nrf2 Translocation and Antioxidant Response in In Vitro Inflammatory Model

Francesco Bellia [1,†], Valeria Lanza [1,†], Irina Naletova [1,†], Barbara Tomasello [2,†], Valeria Ciaffaglione [1], Valentina Greco [3], Sebastiano Sciuto [3], Pietro Amico [4], Rosanna Inturri [4], Susanna Vaccaro [4], Tiziana Campagna [1], Francesco Attanasio [1], Giovanni Tabbì [1,*] and Enrico Rizzarelli [1,3]

[1] Institute of Crystallography, National Council of Research (CNR), P. Gaifami 18, 95126 Catania, Italy; francesco.bellia@cnr.it (F.B.); valeria.lanza@cnr.it (V.L.); irina.naletova@ic.cnr.it (I.N.); valeria.ciaffaglione@ic.cnr.it (V.C.); tiziana.campagna@cnr.it (T.C.); francesco.attanasio@cnr.it (F.A.); erizzarelli@unict.it (E.R.)

[2] Department of Drug and Health Sciences, University of Catania, Viale Andrea Doria 6, 95125 Catania, Italy; btomase@unict.it

[3] Department of Chemical Sciences, University of Catania, Viale Andrea Doria 6, 95125 Catania, Italy; vgreco@unict.it (V.G.); ssciuto@unict.it (S.S.)

[4] Fidia Farmaceutici SpA, Contrada Pizzuta, 96017 Noto, Italy; pamico@fidiapharma.it (P.A.); rinturri@fidiapharma.it (R.I.); svaccaro@fidiapharma.it (S.V.)

* Correspondence: giovanni.tabbi@cnr.it

† These authors contributed equally to this work.

SUPPORTING INFORMATION

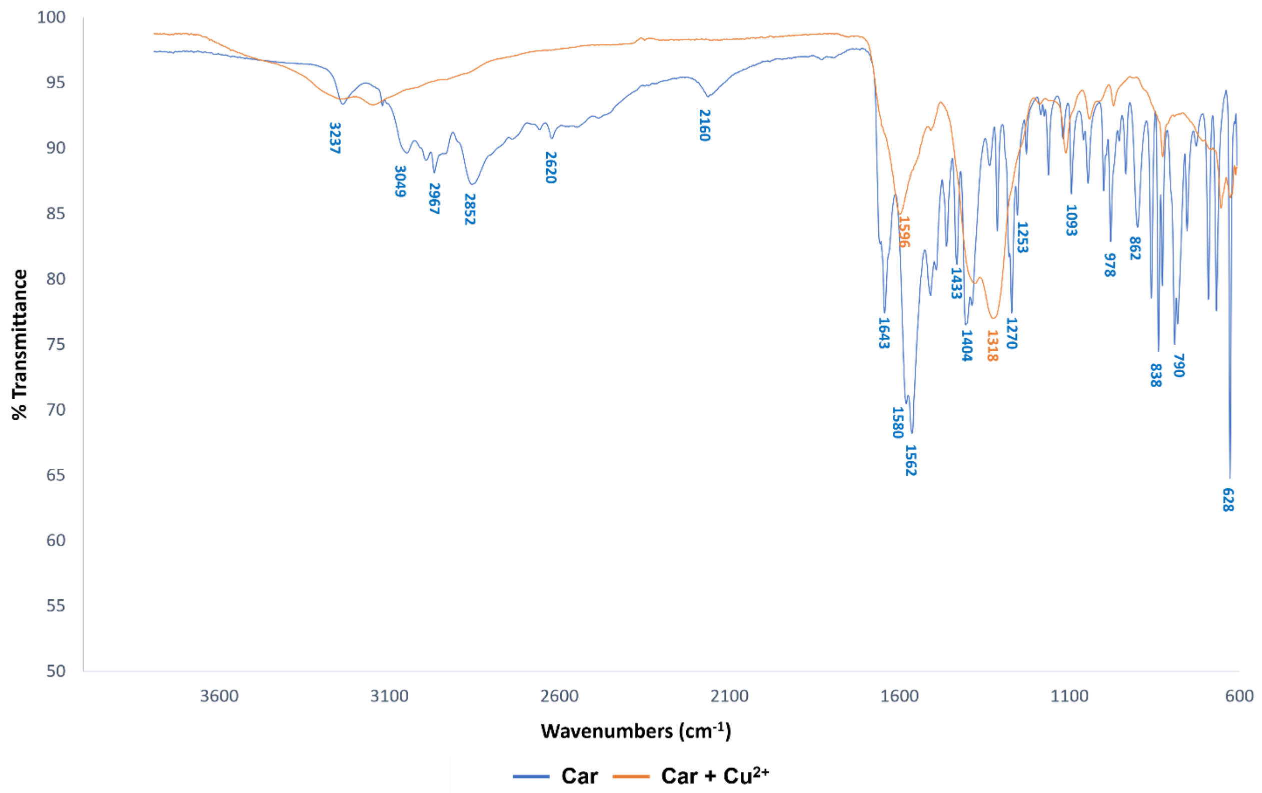

Figure S1. Comparison between FTIR-ATR spectra of Car and its complex with Cu^{2+}. The recorded FTIR-ATR spectrum of Car (blue) shows the characteristic vibrational peaks of the carbonyl, carboxyl, imidazole, and amine groups, as previously reported in the literature [110, 111]. In the presence of Cu^{2+} significant modifications are observed in the FTIR-ATR spectrum of Car (orange). The main changes following complexation with Cu^{2+} involve the amide carbonyl (disappearance of the band at 1643 cm^{-1} related to C=O stretching), the histidine carboxyl (decrease in band intensity at 1404 cm^{-1} and 628 cm^{-1}) and the imidazole ring (disappearance of the band at 1270 cm^{-1}) [111]. Two main bands at 1596 cm^{-1} and 1318 cm^{-1} appear in the presence of Cu^{2+}. Moreover, the vibrations in the high-frequency region, including the stretching modes at 3237 cm^{-1} and 3049 cm^{-1}, for the protonated terminal amine of the free ligand disappear in the complex.

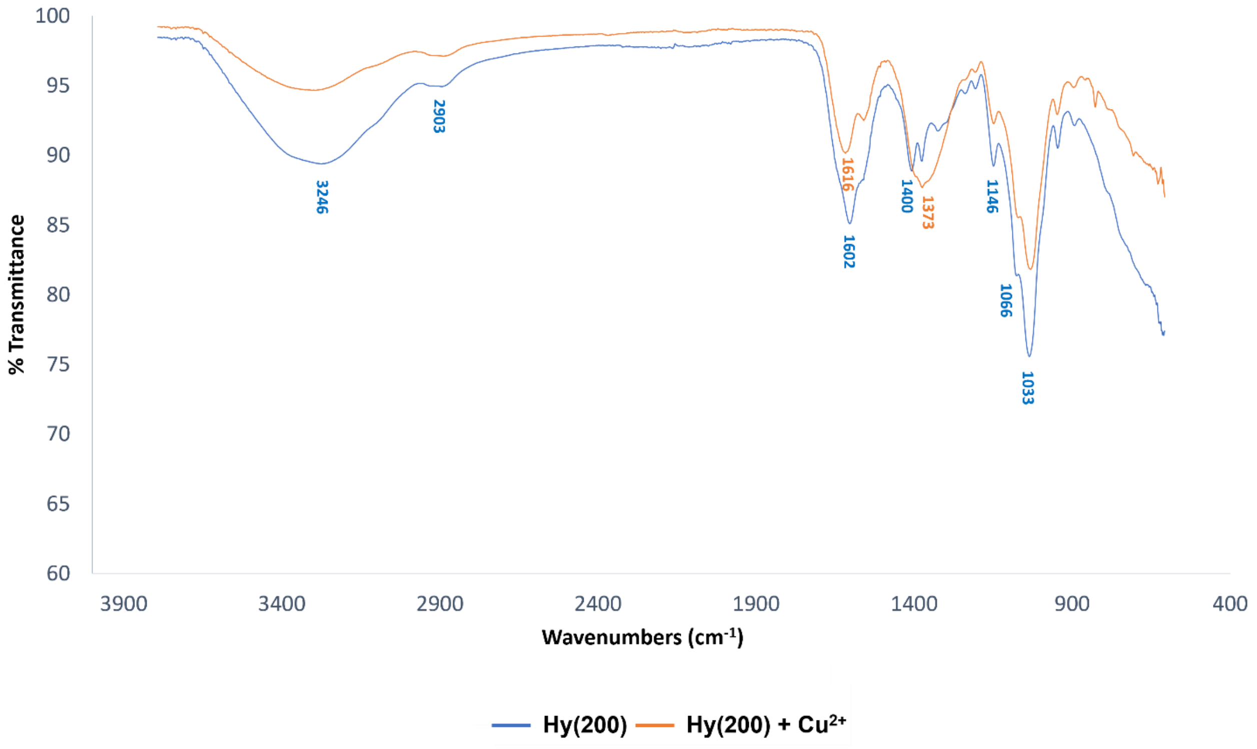

Figure S2. Comparison between FTIR-ATR spectra of Hy(200) and its complex with Cu^{2+}. The main changes observed by comparing the FTIR-ATR spectra of Hy(200) (blue) and the Cu^{2+}Hy(200) complex (orange) involve the reduced intensity of the carboxyl bands at 1602 cm^{-1} and the shift of the band from 1404 to 1373 cm^{-1}, suggesting the binding of Cu^{2+} to the carboxyl oxygen, as previously described [109].

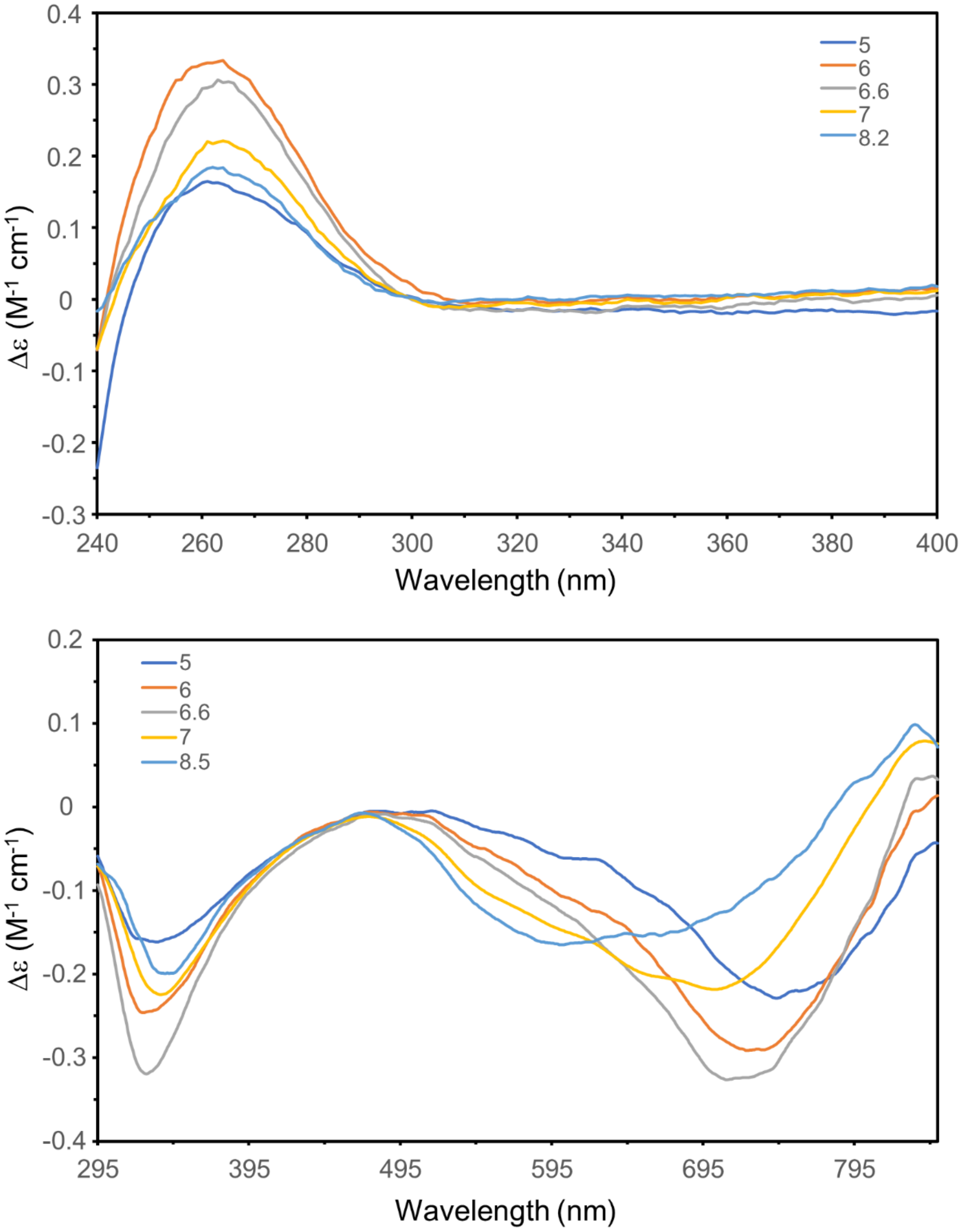

Figure S3. UV-vis CD spectra for Cu^{2+}Hy(200)Car35% at different pH. [HyCar]=8 x 10^{-3} M, [Cu^{2+}]=7 x 10^{-3} M; Upper panel: path length= 0.1 cm, lower panel: path length= 1 cm.

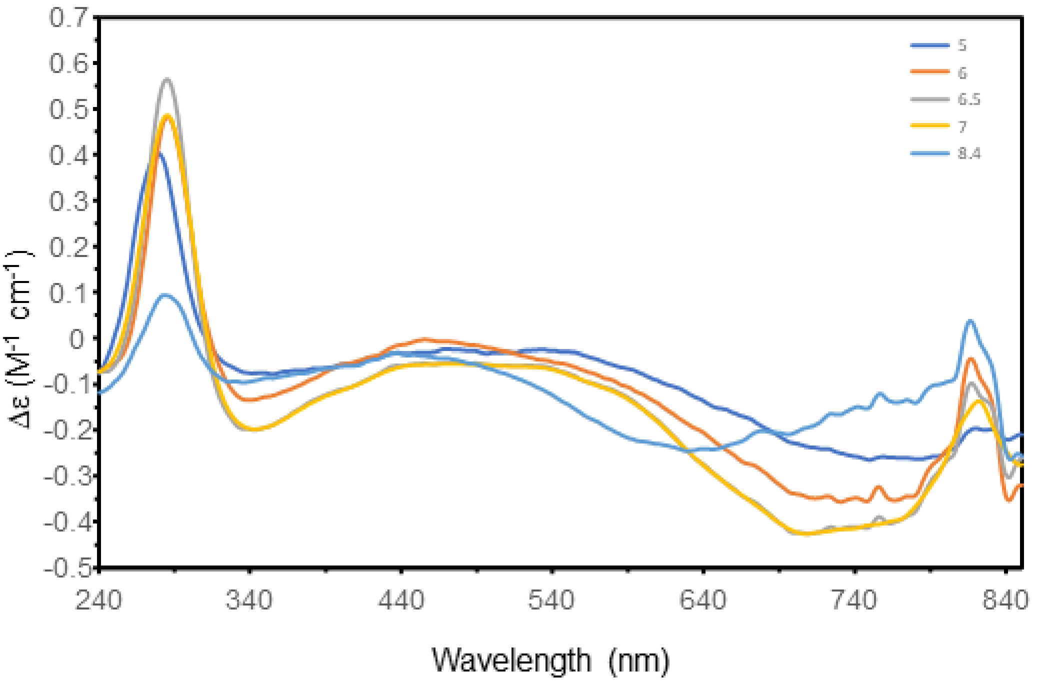

Figure S4. UV-vis CD spectra for Cu^{2+}Hy(200)Car14% at different pH. [HyCar]=3.3 x 10^{-3} M, [Cu^{2+}]=2.7 x 10^{-3} M; path length= 0.1 cm.

Table S1. I_{50} values (indirect method assay) of copper(II) complexes with histidine containing dipeptides and their glycosidic conjugates in phosphate buffer (5-10×10^{-3} mol dm^{-3}, pH 7.4).

SOD1 mimic	I_{50} (μM)	Ref.
Cu–HCar	0.2	b
Cu–TrHCar	0.17	b
Cu–CDAH6	0.24	a
Cu–CDAH3	0.43	a

AH = Carnosine; CD = b-cyclodextrin; Tr = threalose; HCar = homocarnosine; CD derivatives = β-cyclodextrin functionalized with AH at its narrow (CDAH6) or at its wide (CDAH3) rim

[a] Data taken from Ref a) [65], b) [115].

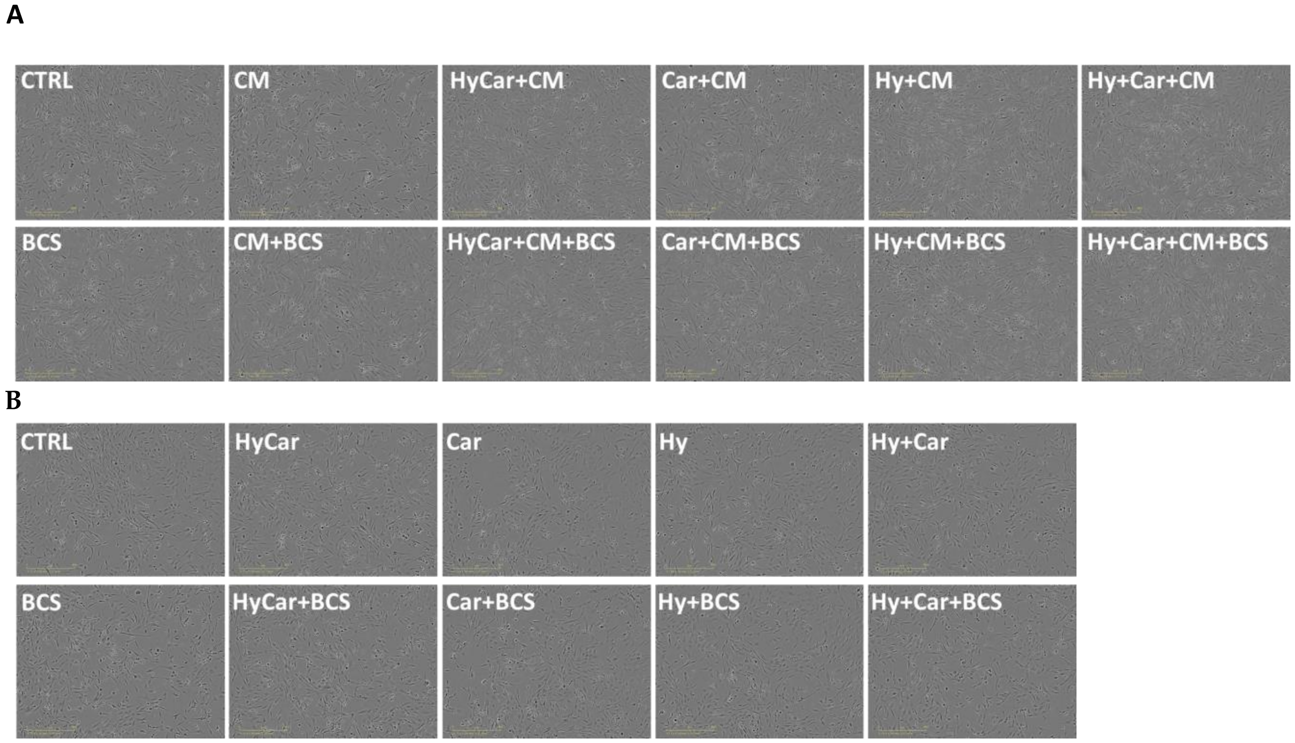

Figure S5. Representative image acquired with the IncuCyte system in brightfield of hFOB cells pre-treated with HyCar, Hy, Car or Hy+Car in the presence or absence of BCS (50 µM) for 1 h and then exposed to MCM **(A)** or to compounds alone **(B)** for 20 h. Scale bar, 400 µm. Magnification, 10X.

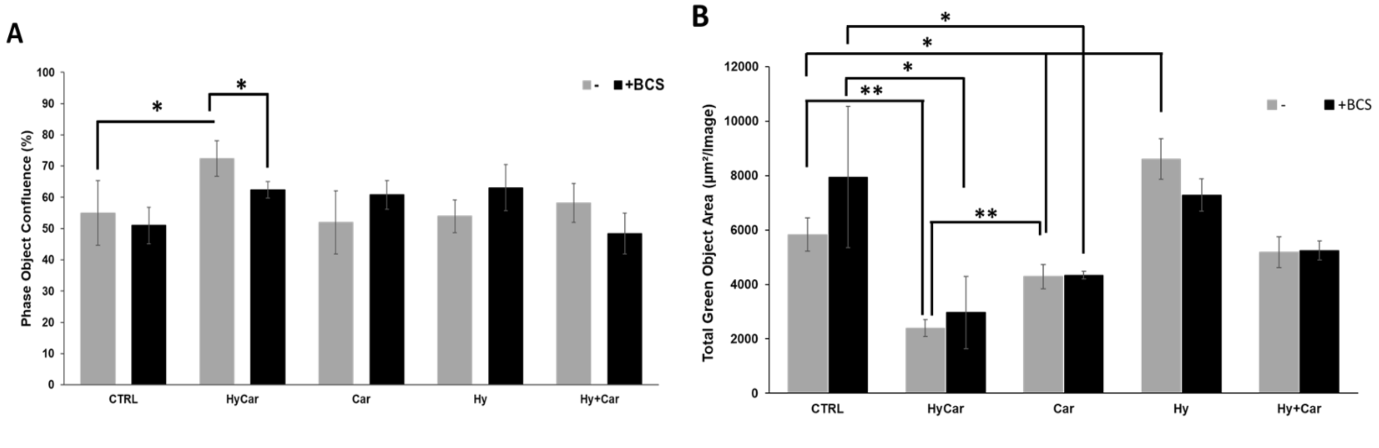

Figure S6. Effect of Hy, Car and their conjugate interaction with Cu^{2+} present in culture medium on cell proliferation **A)** and ROS level in hFOB cells **B)**. hFOB cells were treated with HyCar, Hy, Car or Hy+Car in the presence or in the absence of BCS (50 µM) for 20h. After the treatments, the plates were imaged in the IncuCyte instrument. **A)** Total occupied area of hFOB cells was calculated by IncuCyte Base Analysis with "Artificial Intelligence (AI)" mask for cell detection; for other parameters refer to Table 1 in Material and Methods. The instrument software generated the percentage of cell confluence (as indicated in Materials and Methods) for each well. The data points and error bars represent the mean ± SD of two experiments in quadruplicates. **B)** Fluorescence measurement was performed with the IncuCyte system using the "Artificial Intelligence (AI)" mask for cell detection; for other parameters refer to Table 2 in Material and Methods. Data are expressed as the total green fluorescence area of cells per image. Statistical significance is indicated as *$p<0.05$, **$p<0.005$.

A

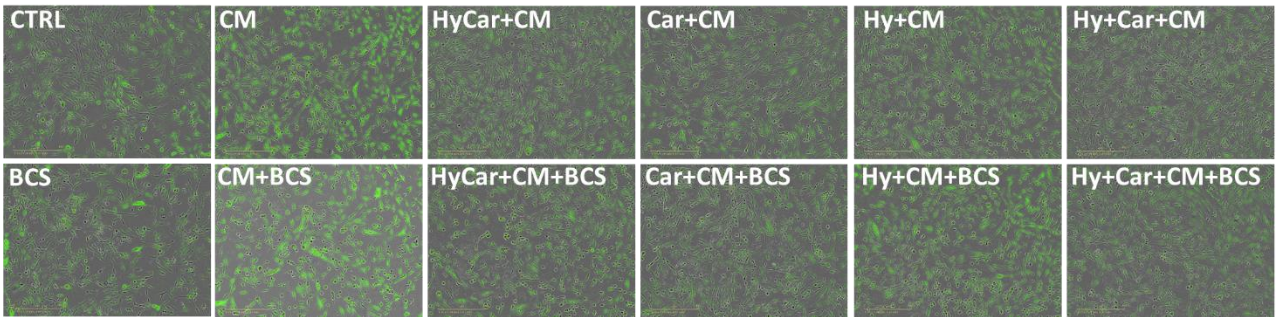

B

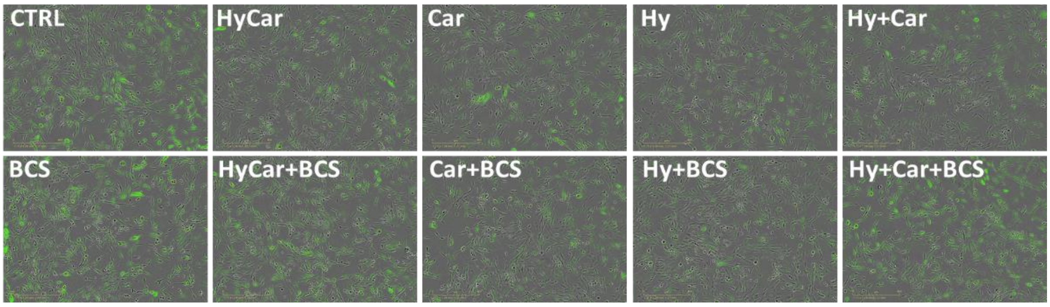

Figure S7. Representative images acquired with the IncuCyte system in brightfield and fluorescence mode (DCF in green, excitation at 485 nm and emission at 530 nm). hFOB cells were pre-treated with HyCar, Hy, Car or Hy+Car in the presence or absence of BCS for 1 h and then exposed to MCM (**A)** and to compounds alone **(B)** for 20 h. Scale bar, 400 μm. Magnification, 10X.

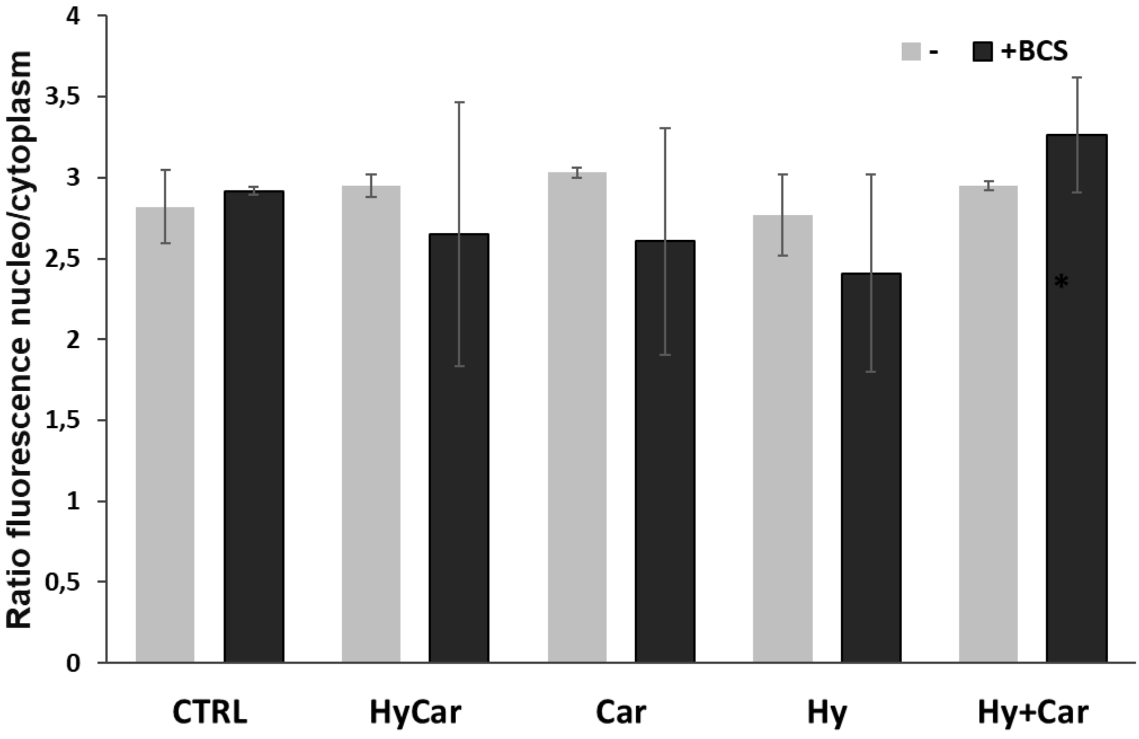

Figure S8. Effect of Hy, Car and their conjugate interaction with Cu^{2+} present in culture medium on nuclear localization of Nrf2 in hFOB. hFOB cells were pre-treated with HyCar, Hy, Car or Hy+Car in the presence or the absence of BCS (50 μM) for 1 h and then exposed to MCM for 48h. Ratio of fluorescence intensity between nucleus and cytoplasm represents the Nrf2 expression level. All values are mean ± SD of 5 randomly field images (see Figure S7) from three independent experiments.

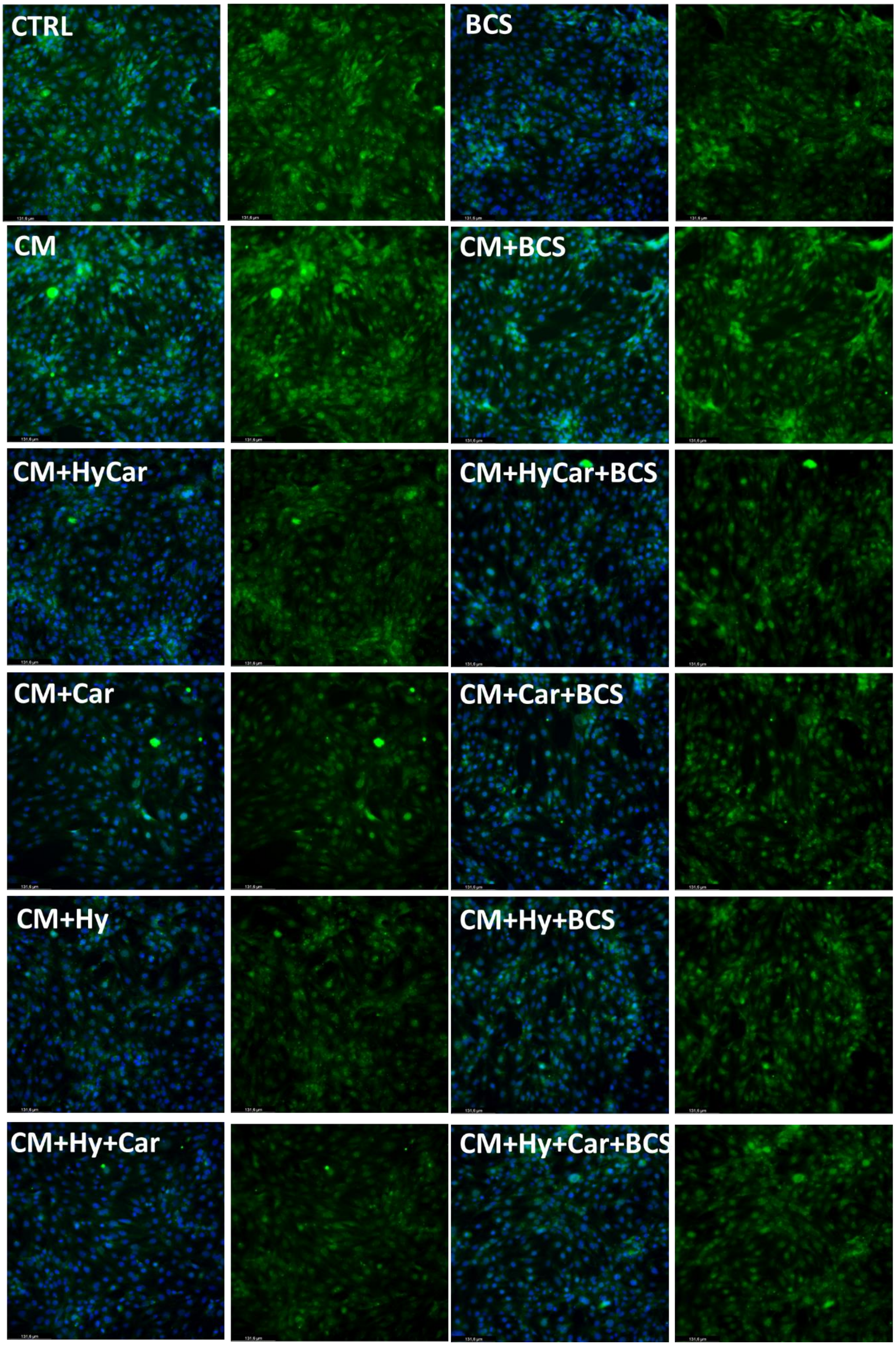

Figure S9. Representative fluorescence image of the nuclear and cytoplasmic expression of Nrf2 in hFOB cells. Average intensity values for the Nrf2 fluorescence corresponding to the Nrf2 protein content in the cytoplasm and nucleus for untreated control and pre-treated cells with HyCar, Hy, Car or Hy+Car mixture in the presence or the absence of BCS (50 μM) for 1 h and then exposed to MCM for 48 h. Magnification 40X. Scale bars are 65.8 μm.

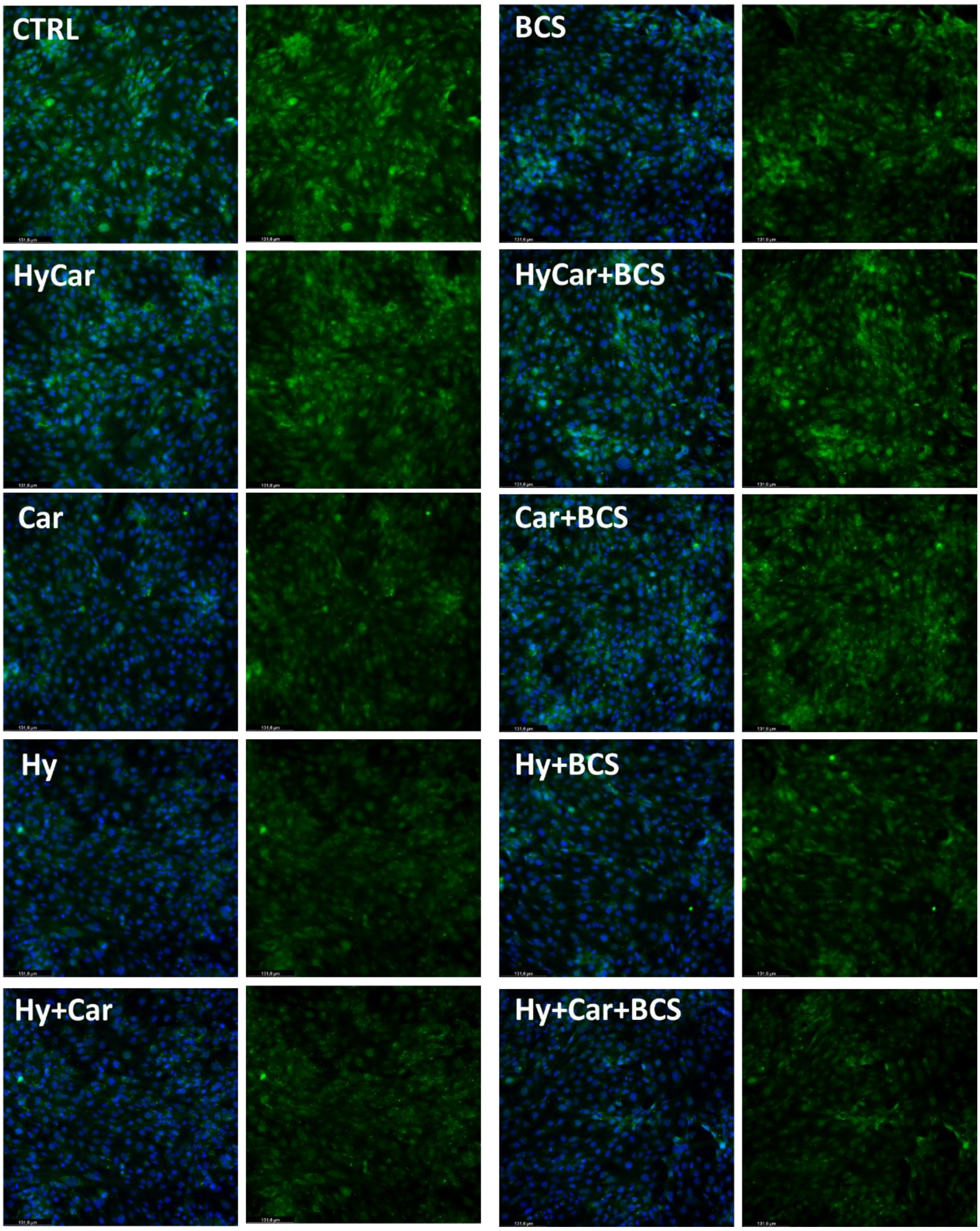

Figure S10 . Representative fluorescence image of the nuclear and cytoplasmic expression of Nrf2 in hFOB cells. Average intensity values for the Nrf2 fluorescence corresponding to the Nrf2 protein content in the cytoplasm and nucleus for untreated control and pre-treated cells with HyCar, Hy, Car or Hy+Car mixture in the presence or the absence of BCS (50 μM) for 1 h and then exposed to compounds alone for 48 h. Magnification 40X. Scale bars are 65.8 μm.

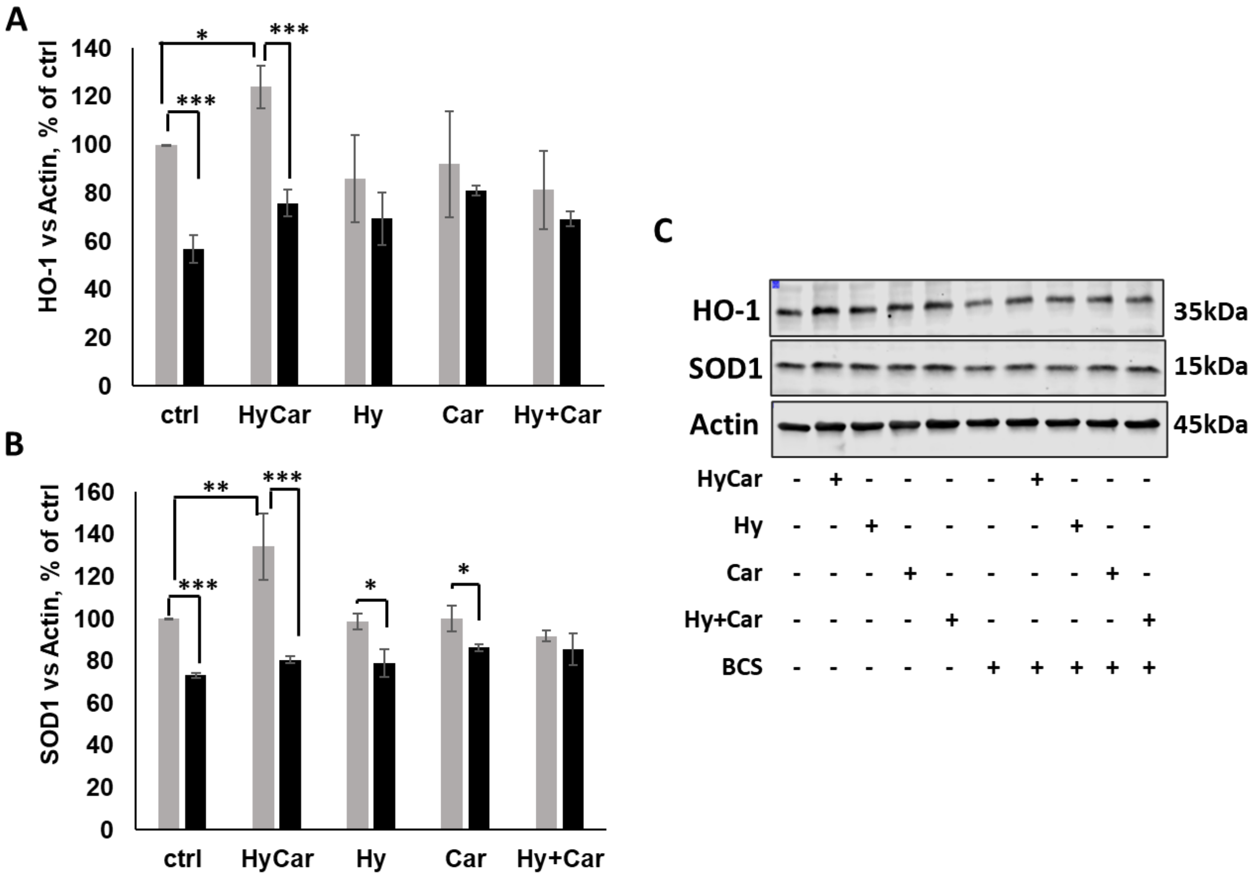

Figure S11. HyCar affects Nrf2 signaling pathway in hFOB cells in copper dependent way. Densitometric analysis (A, B), and representative western blot images (C) of HO-1, SOD1 expression in hFOB treated with HyCar, Hy, Car or Hy+Car in the presence or in the absence of BCS (50 μM) for 49h. The protein expression levels are reported as the ratio over Actin. Data are expressed as mean ± SD. *p≤0.05, **p≤0.01, ***p≤0.001.

References

References [65, 109, 110, 111, 115] are cited in the main text.
